# Silicon and iron nanoparticles protect rice against lead (Pb) stress by improving oxidative tolerance and minimizing Pb uptake

**DOI:** 10.1038/s41598-024-55810-2

**Published:** 2024-03-12

**Authors:** Fozia Ghouri, Samreen Sarwar, Lixia Sun, Muhammad Riaz, Fasih Ullah Haider, Humera Ashraf, Mingyu Lai, Muhammad Imran, Jingwen Liu, Shafaqat Ali, Xiangdong Liu, Muhammad Qasim Shahid

**Affiliations:** 1 State Key Laboratory for Conservation and Utilization of Subtropical Agro-Bioresources, Guangdong Laboratory for Lingnan Modern Agriculture, South China Agricultural University, Guangzhou, 510642 China; 2 Guangdong Provincial Key Laboratory of Plant Molecular Breeding, South China Agricultural University, Guangzhou, 510642 China; 3 Guangdong Base Bank for Lingnan Rice Germplasm Resources, College of Agriculture, South China Agricultural University, Guangzhou, 510642 China; 4 Department of Botany, Government College University, Faisalabad, 38000 Pakistan; 5 Key Laboratory of Vegetation Restoration and Management of Degraded Ecosystems, South China Botanical Garden, Chinese Academy of Sciences, Guangzhou, 510650 China; 6 Department of Environmental Sciences, Government College University, Faisalabad, 38000 Pakistan; 7 Department of Biological Sciences and Technology, China Medical University, Taichung, 40402 Taiwan

**Keywords:** Abiotic, Plant sciences, Environmental sciences

## Abstract

Lead (Pb) is toxic to the development and growth of rice plants. Nanoparticles (NPs) have been considered one of the efficient remediation techniques to mitigate Pb stress in plants. Therefore, a study was carried out to examine the underlying mechanism of iron (Fe) and silicon (Si) nanoparticle-induced Pb toxicity alleviation in rice seedlings. Si–NPs (2.5 mM) and Fe-NPs (25 mg L^−1^) were applied alone and in combination to rice plants grown without (control; no Pb stress) and with (100 µM) Pb concentration. Our results revealed that Pb toxicity severely affected all rice growth-related traits, such as inhibited root fresh weight (42%), shoot length (24%), and chlorophyll b contents (26%). Moreover, a substantial amount of Pb was translocated to the above-ground parts of plants, which caused a disturbance in the antioxidative enzyme activities. However, the synergetic use of Fe- and Si–NPs reduced the Pb contents in the upper part of plants by 27%. It reduced the lethal impact of Pb on roots and shoots growth parameters by increasing shoot length (40%), shoot fresh weight (48%), and roots fresh weight (31%). Both Si and Fe–NPs synergistic application significantly elevated superoxide dismutase (SOD), peroxidase (POD), catalase (CAT), and glutathione (GSH) concentrations by 114%, 186%, 135%, and 151%, respectively, compared to plants subjected to Pb stress alone. The toxicity of Pb resulted in several cellular abnormalities and altered the expression levels of metal transporters and antioxidant genes. We conclude that the synergistic application of Si and Fe-NPs can be deemed favorable, environmentally promising, and cost-effective for reducing Pb deadliness in rice crops and reclaiming Pb-polluted soils.

## Introduction

Heavy metals cause phytotoxicity in crops, even at low levels (i.e., 5–100 μM), and contaminate the environment^1^. The removal of unprocessed industrial wastes is a significant source of heavy metals pollution because of the industrial revolution, especially in pesticide processing factories, metallurgical industries, chemical, paint, acid mine drainage, fertilizers, and leather industries^2,3^. Lead (Pb), nickel (Ni), cadmium (Cd), platinum (Pt), mercury (Hg), antimony (Sb), and copper (Cu) are persistent chemical elements that do not readily degrade in the environment. Consequently, these elements pose environmental challenges, particularly when their bioaccumulation increases, often attributed to human activities^4^. Recently, Pb has been considered a “chemical of very high concern” because it is one of the harmful heavy metal contaminants that linger in the soil for a more extended period^5^. Pb occurs in the bound state or as a free metal ion in soil and ultimately disrupts the microbiota of soil^6^, which enters the soil through several anthropogenic activities, including the use of fertilizers, explosives, paints, pesticides, and batteries^7,8^. In a nationwide survey of agricultural soils, Pb levels surpassed 1.5% of the standard limits set by the Chinese Ministry of Environmental Protection^9^. Le et al.^10^ reported a similar concern, noting that vegetable samples in the vicinity of a bus station exceeded the permitted limit for Pb contamination in Vietnam. In a separate investigation in Bangladesh^11^, Islam et al. found elevated concentrations of Pb, along with Cd, As, Cr, and Ni, in food near industrial zones, surpassing the permitted standards. Permissible levels of Pb in soil are subject to variation based on different guidelines and sources. An acceptable threshold, proposed at 600 ppm of Pb in soil, is considered a “safe” level, contributing no more than 5 µg/dL to the total blood Pb of children under 12 years of age. The natural concentration of Pb in soil exhibits a range from 10 to 40 mg Pb kg^−1^ dry soil, with a median concentration of around 20 mg Pb kg^−1^ dry soil. Soils highly contaminated with Pb may surpass concentrations of 10,000 mg Pb kg^−1^. International standards, such as those defined by the World Health Organization and the Environmental Protection Agency (EPA), set permissible limits for Pb at 0.05 mg L^−1^. Furthermore, the Council of State and Territorial Epidemiologists' (CSTE) blood Pb reference value is established at 3.5 µg dL^−1^, serving to identify individuals with blood Pb levels exceeding the 97.5th percentile among adults and children nationwide.

Pb toxicity disturbs different physiological and biochemical processes in plants, which cause chlorophyll degradation, restrains aquaporins, stunted growth, production of active oxygen species, interferes with ion homeostasis, lipid peroxidation, and nutrient inhibition^12,13^. Reactive oxygen species (ROS) are cleaned up or reduced by a sequence of events that antioxidant enzymes create in plants under Pb stressed environments^14^. Plants have established three main pathways to detoxify the Pb toxicity, including antioxidant defense system activation to remove Pb-induced free ions, an inducible method that is related to detoxification and removal into extracellular spaces, and a passive approach where plants physically block the Pb uptake^15,16^. In rice (*Oryza sativa* L.), the harmful effects of Pb toxicity are evident across various aspects, including a significant reduction in the germination index, root/shoot length, physio-morphological traits, nutrient adsorption through roots, and overall plant yield^15^. Furthermore, Pb adversely affects the activity of ribulose-1,5-bisphosphate in rice, leading to the suppression of CO_2_ fixation. Notably, the presence of 1200 mg kg^−1^ of Pb has been shown to reduce rice yield to 39%. These findings collectively highlight the serious impact of Pb-polluted agricultural soil on food safety. Hence, developing strategies to mitigate Pb-induced toxic effects on plants and the ecosystem is imperative.

Iron (Fe) is a necessary micronutrient and contributes as a cofactor in various biochemical and physiological reactions, which ultimately affect the development and growth of plants^17^. Fe is an insoluble hydrated ferric oxide form in the soil unavailable to plants. Fe is a redox-active transition metal, and plants have adopted sophisticated approaches to fulfill the requirement of Fe in different environments^18^. Silicon (Si) is considered a crucial constituent for plant development and is the 2^nd^ most-ranked element in the atmosphere^19^. Si occurs in the soil solution as monomeric acid or mono-silicic, which plants uptake from the soil by roots. Si contents vary significantly from 0.1 to 10% of the plant's above-ground parts' total dry weight^20^. Previous studies revealed that Fe and Si ameliorate Cd toxicity and help plants absorb soil nutrients in a stressful atmosphere^21,22^.

Previous investigations have demonstrated that nanoparticles (NPs) are critical in various plant processes^23^. Of these NPs, Fe-based NPs are of specific significance because of their magnetic characteristics^24^. Fe–NPs have been broadly applied to clean contaminated water, attracting significant attention from researchers engaged with technologies for treating wastewater^25^. They are commonly used in domestic and industrial sectors, such as agriculture, biomedical, defense, healthcare, food, energy, aerospace, textile, environmental bioremediation, and construction^3,26^. Iron-based NPs are applied as (i) photocatalysts for the alteration of pollutants to non-toxic formulations; and (ii) absorbent or immobilization carriers^27^. Different types of pollutants, such as explosives, inorganic ions, pesticides, toxic metals, and chlorinated compounds, are targeted by Fe-based NPs^28^. Fe-NPs have been strategically applied to remediate pollutants, notably Cd^29^ and As^30,31^, in regions where wheat (*Triticum aestivum* L.) and rice are cultivated under metal-contaminated soils. Applying silica NPs resulted in reduced metal intake and enhanced the biological yield of rice crops^32^. Si fertilizers have low bioavailability, and using Si-NPs could be the paramount choice for Si accumulator species, including rice and wheat^33,34^. In maize (*Zea mays* L.), Si-NPs (10 μM) significantly alleviated As stress (50 μM) compared to Si alone (10 μM). This enhanced efficacy of Si-NPs may be attributed to their better accessibility, in contrast to common silica, under the experimental conditions^35^.

Recently, nanotechnology has been extensively applied in numerous regions, such as agriculture and industries^33,36,37^. Substantial evidence has revealed that plant development is affected by Fe-NPs. Still, the overall response of plants against different levels of NPs largely depends on plant species, the extent of exposure, and growing media^38^. Previous studies have revealed that Fe-NPs play a more positive role in plants' physiological and morphological characteristics than Fe fertilizer^39,40^. Fe–NPs (1–20 ppm) positively affect lettuce seeds' root length and germination^41^. Applying citric acid-coated Fe_3_O_4_ NPs (20 ppm) for five days greatly enhanced enzyme activities and did not distress wheat's lipid peroxidation and growth in the hydroponic culture^42^.

Thus, Fe and Si–NPs have excellent prospects for remediating metal-polluted soils. Further research is required to check the significance of NPs on heavy metal detoxification in plants. Though few previous studies have revealed that Fe and Si-NPs can decline the Pb uptake in plants, more information is still necessary to know the combined effect of both NPs in reducing the Pb uptake by rice. Additionally, previous investigations focused on the role of Si-NPs in reducing Pb accumulation in various plant species, including wheat, rice, and maize^33,35,41^. The combined application of Fe and Si nanoparticles holds the potential for greater effectiveness compared to using either nanoparticle alone due to their synergistic effects. Meanwhile, Fe-NPs contribute to enhanced nutrient availability and oxidative stress mitigation. Similarly, Si-NPs are known for improving plant resilience and stress tolerance. However, despite the potential benefits of these individual NPs, the exploration of their combined application to mitigate Pb toxicity effects in rice remains an unexplored research gap. Investigating the synergistic effects of Fe and Si-NPs in addressing Pb toxicity could provide valuable insights into developing more effective strategies for enhancing plant resistance and mitigating the adverse impacts of heavy metal stress on rice. Therefore, this work was planned to check the efficiency of the synergistic application of Si and Fe–NPs in decreasing Pb accumulation in rice and their effects on antioxidant enzymes, chlorophyll contents, plant growth, and oxidative stress parameters. We assume that applying Fe- and Si–NPs can overcome Pb stress by enhancing the enzymatic activities and decreasing oxidative stress, thus reducing Pb uptake in rice.

## Materials and methods

### Plant materials and growth husbandry

The rice seeds of an inbred line, DW-4, (collected from our germplasm resource at South China Agricultural University (SCAU), Guangzhou, China) were surface-sterilized for about 10 min in 5% v/v NaOCl solution and soaked for 24 h in the ddH_2_O after rinsing with distilled water. The plant materials utilized in the present study conform to relevant national or institutional guidelines. Then, seeds were held in a growth chamber on the cheesecloth for germination for a week, and identical seedlings were shifted to pots (12 seedlings/pot) filled with two liters of one-quarter strength nutrient solution by 1/2-strength and full-strength nutrient solution in the next week. A hydroponic experiment was done under controlled conditions in a growth chamber at SCAU with a 28 °C temperature at 65–70% relative humidity and a 10/14-h dark/light cycle. The hydroponic culture was consisted of $1/2$ kimura nutrient solution; 0.36 mM Ca(NO_3_)_2_.4H_2_O, 0.36 mM (NH_4_)_2_SO_4_, 0.55 mM MgSO_4_.7H_2_O, 0.27 mM K_2_SO_4_, 9.10 µM MnCl_2_, 0.77 µMZnSO_4_.7H_2_O, 0.15 µM (NH_4_)6Mo_7_O_24_.4H_2_O, 0.18 mM KH_2_PO_4_, 20 µMH_3_BO_3_, and 0.32 µMCuSO_4_.7H_2_O. For Fe-deficient treatment, $1/2$ Kimura solution containing no Fe(II)-EDTA was applied, while $1/2$ Kimura solution was applied for Fe-normal treatment. All nutrient solutions were changed every three days, and pH was maintained at 6.5 ± 0.1 with NaOH or HCl.

The Fe-NPs were purchased from Pantian Nanomaterials Co., Ltd. (Shanghai, China), providing Fe_2_O_3_ NPs (99.9%, 20e30 nm), which have been characterized via SEM (scanning electron microscope) (V 460, FEI). SEM observation revealed that nano-oxide iron materials are rod-like and distributed in tight clusters (Fig. S1A). The distance between the long and short axes is around 50–150 nm and 15–25 nm, respectively. Our samples' FTIR (Fourier-transform infrared spectroscopy) spectra revealed that the FeO was highly pure (Fig. S1B). XRD (X-ray diffraction) patterns matched up with conventional X-ray diffraction cards (Fig. S1C). Here, we applied Si-NPs (purchased commercially available) with 99.9% purity exemplified via FTIR, SEM, and XRD of the Fe-NPs. The size distribution showed that SiO_2_-NPs were uniformly distributed with particle sizes of Si-NPs between 15 and 25 nm and exhibited particular aggregation (Fig. S1D). The XRD showed that Si-NPs lacked contaminants and were amorphous, including SiO_2_. Moreover, no additional peaks were discovered. The FTIR spectrum and spectrum pattern confirmed that the material's main component was SiO_2_, validating the correctness of the material used (Fig. S1E). FTIR analysis of Si-NPs revealed that the surface of the nanoparticles and their functional groups contained Si–O–Si, –COOR, and -OH functional groups. The energy spectrum analysis showed that the wt% of Si in the nanomaterial is 71.57%, and the wt% of O is 28.43%, revealing that Si-NPs were amorphous and free of contaminants (Fig. S1F).

Lead (Pb) was applied as Pb(NO_3_)_2_ at the rate corresponding to 100 µM concentrations in the solution. In contrast, Si–NPs and Fe–NP were applied at 2.5 mM and 25 mgL^−1^, respectively. The Pb, Si, and Fe–NPs doses were standardized based on trial experiments, and the treatments were applied to the plants after a 14-day growth period in hydroponic culture. Each treatment combination consists of four pots of replications, and the investigation was conducted in a completely randomized design (CRD). After growing in hydroponic culture, 24-days-old seedlings were removed from the solution. The metal absorption was assessed from the shoot and roots of rice seedlings. Moreover, some fresh samples were preserved in the refrigerator at − 80 °C for qPCR and evaluation of enzymatic activities.

### Evaluation of plant biomass production

The plants were picked ten days after treatment application. The plant samples were separated into above- and below-ground portions. Fresh biomass of below- and above-ground plant parts was estimated. Shoot and root samples were dried at 70 °C for four days, and the dry biomass of above- and below-ground components was measured by ordinary weight balance.

### Analyses of carotenoid and chlorophyll contents

About 0.5 g of fresh rice leaf tissues were sliced and placed in 80% chilled acetone for 24 h. Then, the mixture was centrifuged for 10 min at 5000 rpm (4 °C). The contents of chlorophyll b, carotenoids, and chlorophyll a were observed spectrophotometrically (Jenway 6400, London, UK) in the supernatant at wavelengths 479 nm, 663 nm, and 645 nm, respectively^43,44^.

### Measurements of Pb concentration in rice seedlings

The Pb contents from roots and shoots of rice seedlings were estimated. First, we carefully rinsed the samples with ultra-high pure ddH_2_O and later dried these samples at 60 ºC until constant weight. Plant samples of about 0.2 g were measured, and HNO_3_-HClO (V: V = 6:2) was placed into a Teflon bottle with plant samples and heated on a hotplate at 180 ºC until 1 mL liquid was left in the tube. Subsequently, samples were diluted, and the final quantity of Pb in the solution was estimated by an Agilent 7700 inductively coupled plasma mass spectrometry (ICP-MS) (Agilent Technologies, CA, USA).

### Cytological investigations by transmission electron microscope (TEM)

Cell structure was observed by TEM as described previously^45^. The root tip samples were cut and prefixed in 2.5% glutaraldehyde and then post-fixed in 2% OsO_4_ for two hours. Then, specimens were rinsed with PBS, and a series of ethanol solutions with diverse concentrations (30, 50, 70, 80, 90, and 100%) were used to dehydrate and fix in spur resin. Then, a microtome (Leica, Germany) was used to cut ultrathin sections. The cell structure was seen under a TEM equipped with an Oxford INCA Energy TEM 200 EDX system (Philips TECNAI 10, Netherlands).

Semi-thin sections of rice plant roots were made using semi-thin section kits (Technovit7100). The root samples were immersed in FAA solution for 24 h before being dehydrated with different ethanol concentrations^44^. After that, we treated the samples with a semi-permeable agent at 4 °C for 12 h before immersing them in the pure penetrating agent. After solidification, the material was polymerized at 23–37 °C, and slices (4 µ) were cut from embedded blocks using a thin rotary slicer (Leica RM2265) and inspected and photographed using an automatic biomicroscope (Motic BA310).

### qRT-PCR analysis

The rice samples were collected after treatments, and RNA Kit was used to isolate total RNA. The NanoDrop spectrophotometer was used to determine the purity of the RNA samples. One µg of RNA was taken to prepare the cDNA library from total RNA with a reagent kit. The primers for each gene were developed by primer 5 software, and all primers' sequences are listed in Table S1. Using a Bio-Rad real-time PCR system and SYBR Green reagent (Takara, Kyoto, Japan), qRT-PCR was performed. Three biological replicates were used for each treatment to quantify the expression levels. Actin was an internal control in the 2^-△△Ct^ approach used to estimate the relative expression patterns^46^. We calculated the relative expression using the formula F = 2^^-ΔΔCt^, where ΔΔCt = (CT,Target—CT,Act) Ex—(CT,Target—CT,Act) CK. Ex means experiment group, while CK means control.

### Antioxidant and oxidant activities

Antioxidative and oxidant activities, including CAT (catalase), POD (peroxidase), GSH (glutathione), MDA (malondialdehyde), SOD (superoxide dismutase), and H_2_O_2_ (hydrogen peroxide) were estimated using commercially available kits^44^.

### Statistical analyses

The data, from three independent replications, underwent one-way ANOVA analysis using the SPSS 20.0 program and Graph Pad Prism (version 8.0.2). Bar graphs illustrating the mean and SE for each parameter were constructed. The Tukey test was employed to estimate the least significant difference (LSD) at *p* < 0.05. Mean values and standard deviations (± SD) from four replications were used for representation. Additionally, using R software, a comprehensive exploration of the data's multivariate patterns was conducted through principal component analysis (PCA). Significance between treatments was determined via an LSD test at a 95% probability level.

## Results

### Plants biomass and Chlorophyll

**Figure 1:**
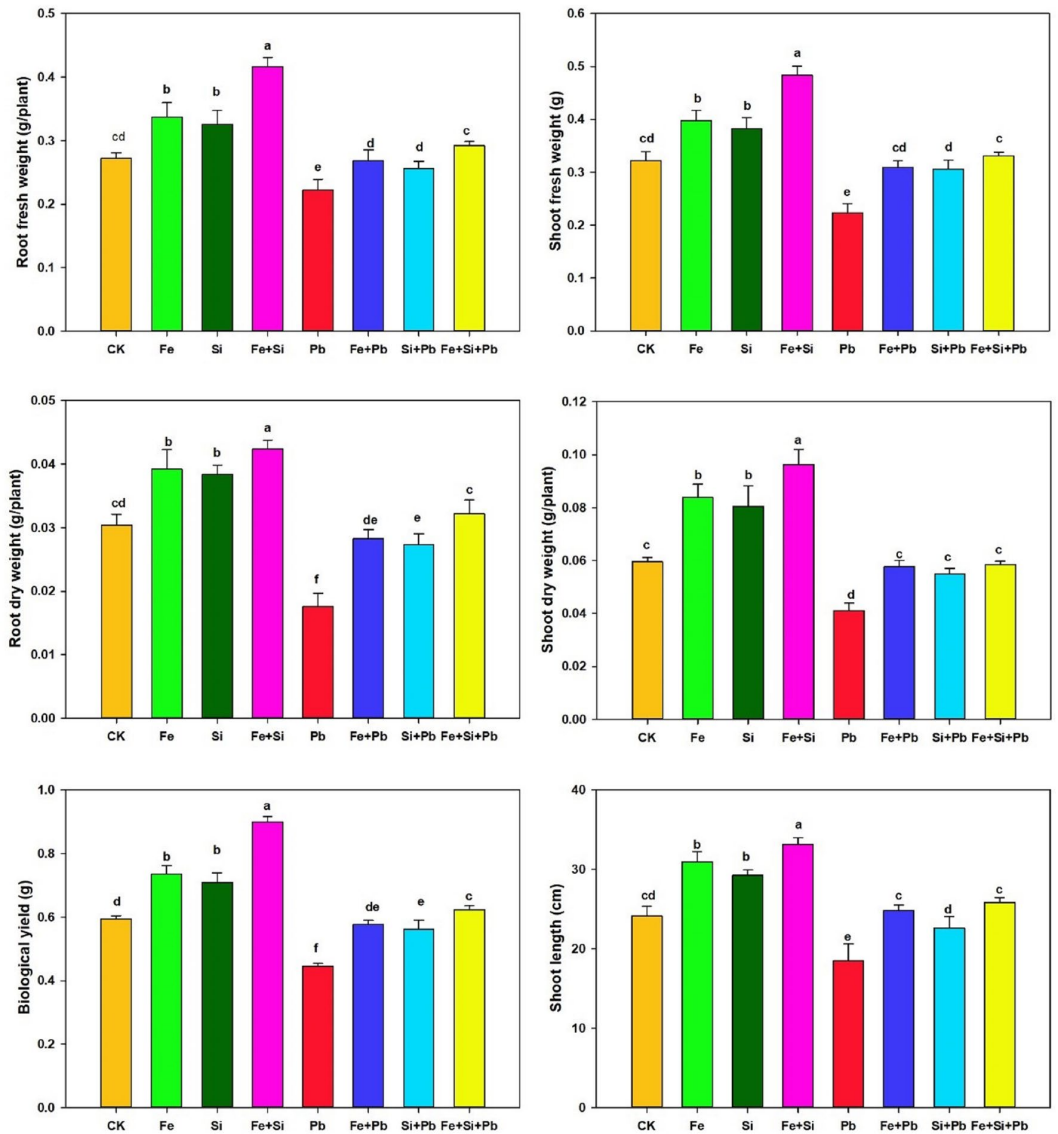
Pb-stressed rice seedling growth response to Si and Fe nanoparticles. Small letters above the bar show the significant difference, and results are denoted by the LSD test and mean ± SD at *p* ≤ 0.05.

We investigated various growth parameters, viz., root fresh weight (RFW), shoot dry weight (SDW), shoot fresh weight (SFW), root dry weight (RDW), and shoot length (SL), as shown in Fig. 1. The results indicated that Pb exposure decreased the SL of rice plants by 23.49% compared to the control plants and RFW, SFW, SDW, and RDW by 18.21%, 30.58%, 30.92%, and 42.10%, respectively. But, the Pb-stressed plants treated with Si and Fe-NPs revealed better performance for all the aforementioned agronomic traits than Pb-stressed plants. Overall, the alone treatment of Fe- and Si-NPs resulted in a much lower increase in all morphological features compared to the combined application of both nanoparticles under a Pb-stressed environment (Fig. 1; Table 1). However, the combined application of Fe- and Si-NPs to the Pb-stressed rice plants increased SL, SFW, RFW, SDW, and RDW by 39.78%, 48.23%, 31.26%, 42.33%, and 82.95% as compared to Pb alone, respectively. These outcomes exhibited the constructive influence of Si- and Fe-NPs in improving Pb resistance in rice (Table 1).

**Table 1:** The findings of the 2 × 2 × 2 factorial ANOVA show the impact of Pb, Silicon, and Iron nanoparticles on different parameters.

Variables	Fe	Si	Pb	Fe × Si	Fe × Pb	Si × Pb	Fe × Si × Pb
SL	< 0.001	< 0.001	< 0.001	< 0.001	0.461	0.087	0.065
RFW	< 0.001	< 0.001	< 0.001	< 0.001	0.757	0.178	0.086
RDW	< 0.001	< 0.001	< 0.001	< 0.001	0.134	0.033	0.213
SDW	< 0.001	< 0.001	< 0.001	< 0.001	0.568	0.149	0.74
Pb root	0.988	0.979	< 0.001	0.974	< 0.001	< 0.001	< 0.001
Pb shoot	0.293	0.071	< 0.001	0.051	< 0.001	< 0.001	< 0.001
Fe root	< 0.001	0.524	0.464	< 0.001	< 0.001	0.66	< 0.001
Fe shoot	< 0.001	0.855	0.014	< 0.001	< 0.001	0.314	< 0.001
Si root	0.858	< 0.001	0.699	< 0.001	0.697	< 0.001	< 0.001
Si shoot	0.977	< 0.001	0.853	< 0.001	0.844	< 0.001	< 0.001
SOD	< 0.001	< 0.001	< 0.001	< 0.001	< 0.001	< 0.001	< 0.001
POD	< 0.001	< 0.001	< 0.001	< 0.001	< 0.001	< 0.001	< 0.001
CAT	< 0.001	< 0.001	< 0.001	< 0.001	< 0.001	< 0.001	< 0.001
GSH	< 0.001	< 0.001	< 0.001	< 0.001	< 0.001	< 0.001	< 0.001
Chl a	< 0.001	< 0.001	< 0.001	< 0.001	0.643	0.674	0.001
Chl b	< 0.001	< 0.001	< 0.001	< 0.001	0.005	0.032	0.001
Carotenoids	< 0.001	< 0.001	< 0.001	< 0.001	0.002	0.003	< 0.001
H_2_O_2_	< 0.001	< 0.001	0.001	< 0.001	0.201	0.302	0.002
MDA	< 0.001	< 0.001	< 0.001	< 0.001	< 0.001	< 0.001	< 0.001

The Iron nanoparticles in root (Fe Root), shoots (Fe Shoot), Si nanoparticles contents in roots (Si Root), shoots (Si Shoot), Pb contents in roots (Pb Root), shoots (Pb Shoot), RFW (root fresh weight), SDW (shoot dry weight), RDW (root dry weight) malondialdehyde (MDA), superoxide dismutase (SOD), shoot length (SL), chlorophyll a (Chl a), Peroxidase (POD), chlorophyll b (Chl b), root fresh weight (RFW), carotenoids (CAR), glutathione (GSH), catalase (CAT), shoot Length (SL), and hydrogen peroxide (H_2_O_2_).

The results indicated that Pb exposure decreased carotenoids and chlorophyll a, b contents by 19.19%, 20.50%, and 25.73% in comparison to the control treatment, respectively (Fig. S2). However, the exclusive utilization of Si and Fe-NPs resulted in significant enhancements in the levels of chlorophyll a (27.03% and 24.67%), chlorophyll b (54.57% and 49.49%), and carotenoid (34.29% and 33.46%) under Pb-induced stress, respectively. The synergistic application of Fe- and Si-NPs to Pb-stressed rice plants increased chlorophyll a, b, and carotenoids by 35.48%, 58.57%, and 37.65% compared to Pb alone treatment, respectively.

### Si and Fe-NPs reduced Pb uptake to mitigate Pb toxicity

**Figure 2:**
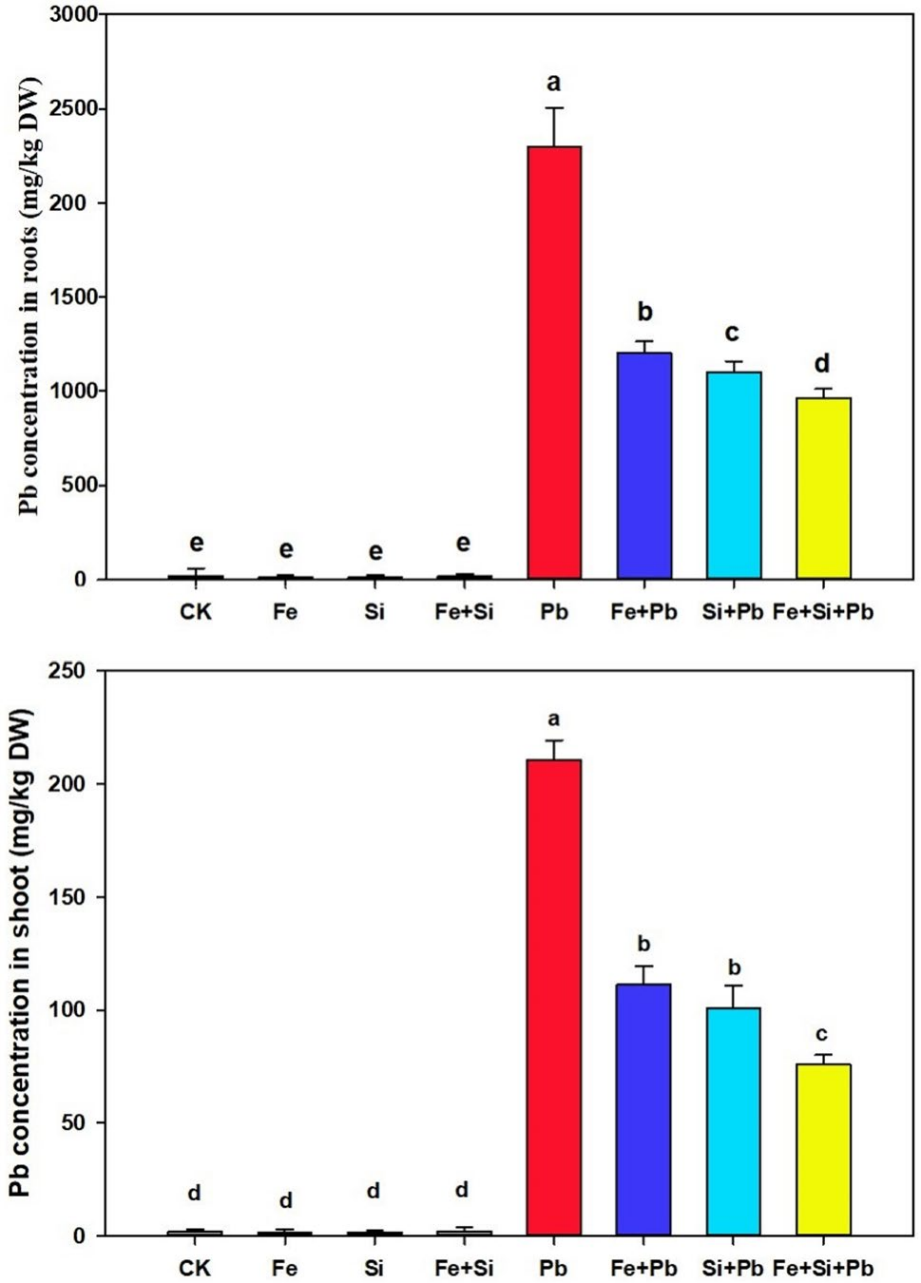
Concentrations of Pb in rice seedlings under Si and Fe nanoparticles. Small letters above the bar show the significant difference, and results are denoted by the LSD test and mean + SD at *p* ≤ 0.05.

The results indicated that both Si and Fe nanoparticle application in the rice seedlings increased their concentrations in rice plants' tissues (shoot and root). The highest contents of Si and Fe were detected in the roots than in the shoots (Fig. S3). The plants exhibited a notable increase in Pb concentration when subjected to Pb application, as compared to non-stressed plants (Fig. 2). Applying Fe and Si-NPs to Pb-stressed plants significantly decreased Pb concentration in shoot and root samples as opposed to Pb -alone treated rice plants. Our results revealed that Pb decreased by 42.94% and 48.31% under Fe- and Si–NPs treatments in the root, and by 11.08% and 13.08% in shoots relative to Pb -alone treated plants, respectively (Fig. 2). Moreover, the joint application of Fe- and Si–NPs had a more noticeable impact on reducing Pb quantity in roots and shoots, and decreased Pb by 56.43% and 26.86% compared to Pb-stressed plants alone, respectively (Fig. 2).

### Effect on antioxidant and oxidant activities

**Figure 3:**
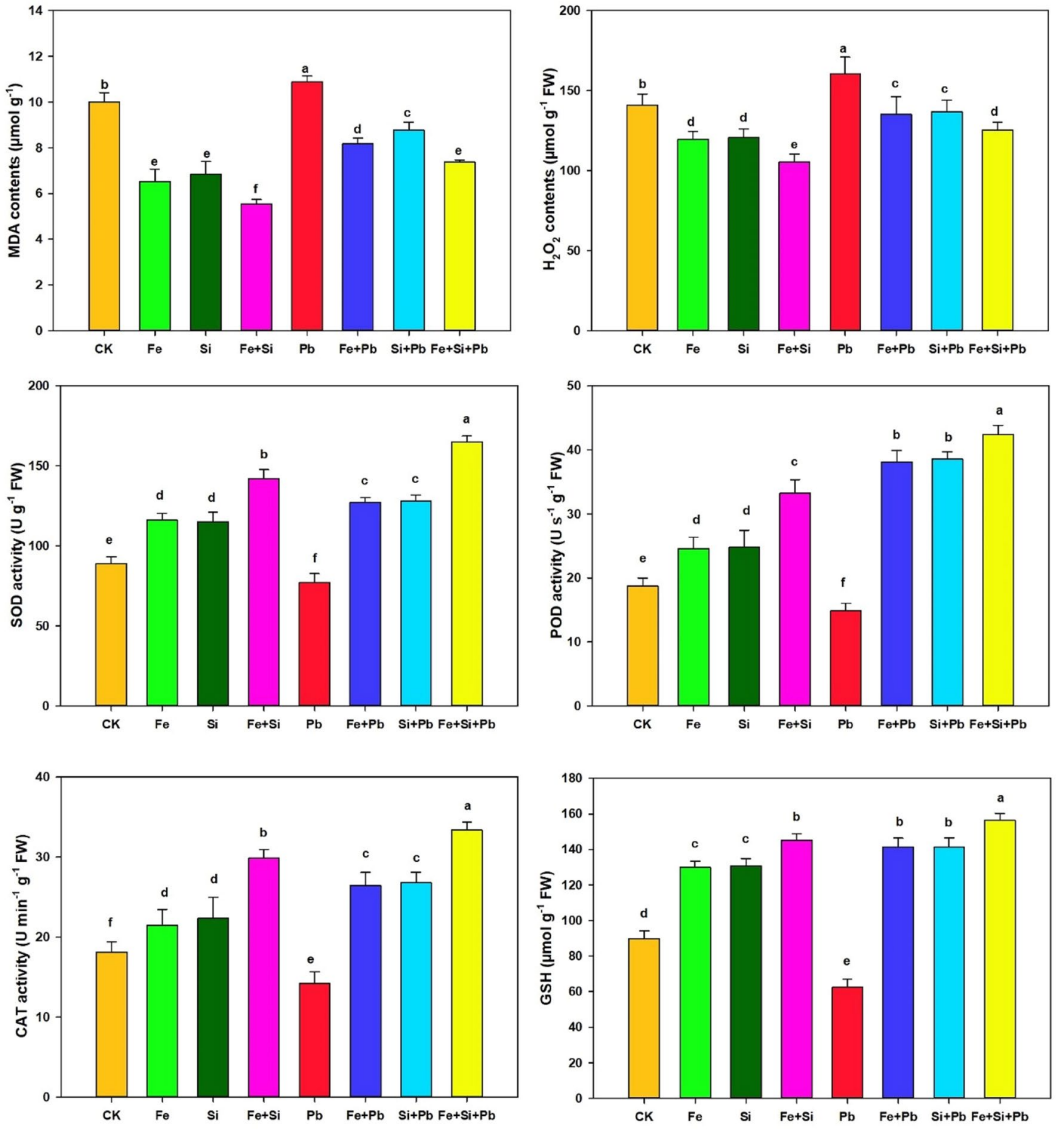
Response of antioxidant system in rice seedlings under Si and Fe nanoparticles. Small letters above the bar show the significant difference, and results are denoted by the LSD test and mean + SD at *p* ≤ 0.05.

Upon subjecting rice seedlings to Pb application, the levels of ROS, including MDA and H_2_O_2_, experienced increases of 8.59% and 13.74%, respectively. Remarkably, the application of Fe-NPs, Si-NPs, and their combined usage resulted in substantial enhancements in SOD activity, with increments of 64.93%, 66.00%, and 114.32%. Furthermore, the activities of POD, CAT, and GSH displayed notable increases. Specifically, Fe-NP treatment led to increments of 156.77%, 85.77%, and 126.58%, while Si-NP treatment resulted in elevations of 159.77%, 88.59%, and 126.34%, respectively. The combined application of both nanoparticles showed even more pronounced effects, with increments of 185.51%, 134.75%, and 150.49% for POD, CAT, and GSH, compared to plants stressed by Pb alone (Fig. 3).

### Influence of Si and Fe-NPs on root ultrastructure

**Figure 4:**
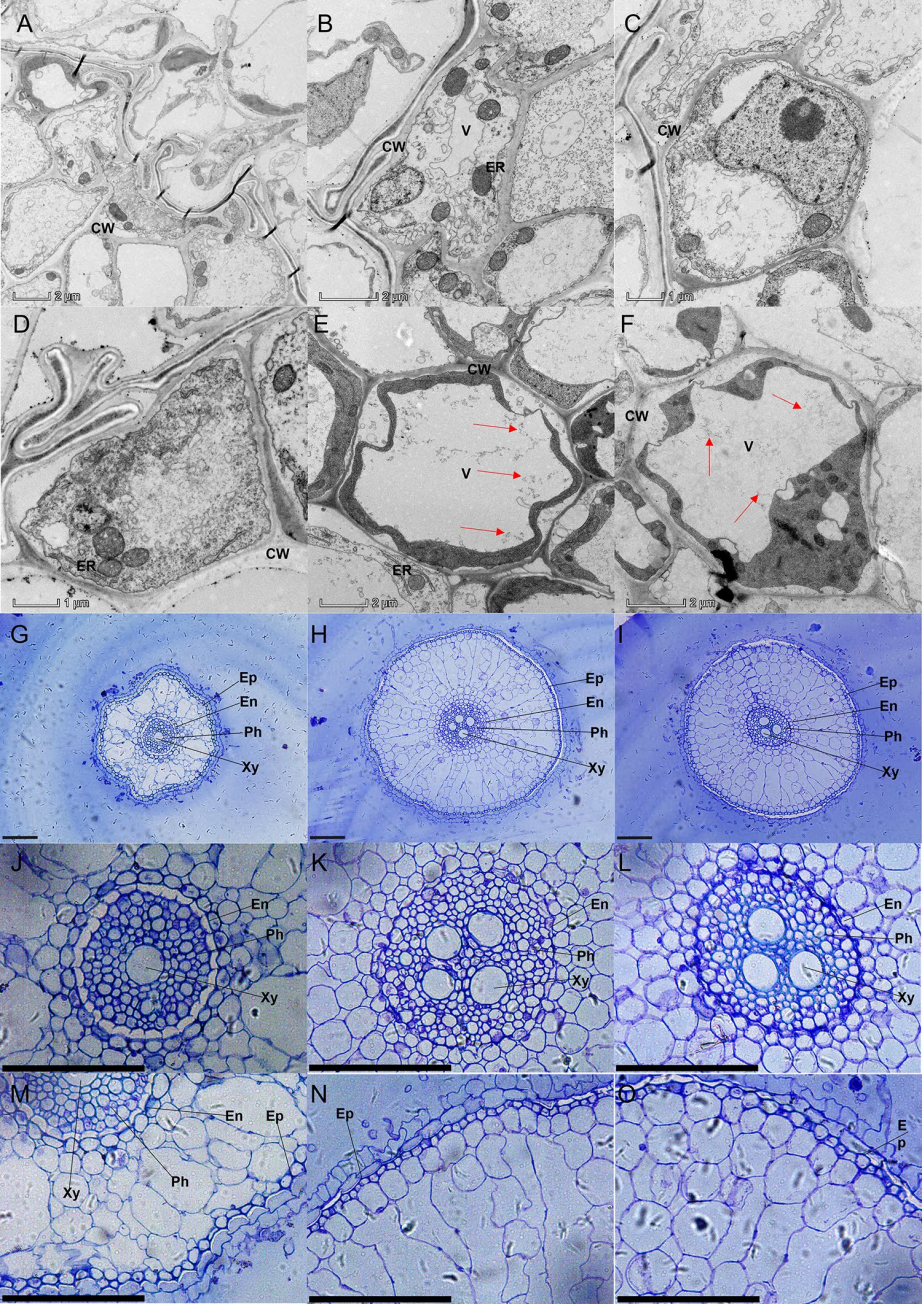
Ultrastructure of root cells of rice under Pb exposure and combined use of Si and Fe nanoparticles. Ultrathin sections of rice seedlings treated with Pb (**A**–**D**), Si + Fe (**E**), and Si + Fe + Pb (**F**) were observed by TEM. Semi-thin section observation under the application of Pb (**G**, **J**, **M**), Si + Fe (**H**, **K**, **N**), and Si + Fe + Pb (**I**, **L**, **O**). CW: Cell wall; V: Vacuole; ER: Endoplasmic reticulum; Xy: Xylem; Ph: Phloem; En: Endodermis; Ep: Epidermis

The root cortex has a well-maintained cell structure with fine-shaped vacuoles and cell wall under non-stressed conditions. The cell organelles were distorted with disordered and swollen vacuoles under Pb-stressed plants (Fig. 4). In the Pb treatment, cell arrangement was disordered. By contrast, Si and Fe–NP-treated plants retained cell morphology, although some displayed distortion. Compared with Pb-stressed plants, vacuoles, cell wall, and mitochondria were closely organized in Fe- and Si–NPs treated plants. Our results showed that cortical cells breached under Pb exposure, while Fe- and Si–NPs treated plants under Pb exposure-maintained vacuole structure and cell wall that sustained the cell integrity. Semithin sections of only Pb-treated plants revealed anomalies in the pericycle, epidermis, endodermis, vascular cylinder, and cortex. In contrast, co-application of Fe and Si under Pb exposure revealed fewer abnormalities (Fig. 4). Normal cell structure was seen in rice plants treated with Fe- and Si–NPs.

### Gene expression levels of antioxidant and metal transporter genes under Si and Fe–NP and Pb stress

**Figure 5:**
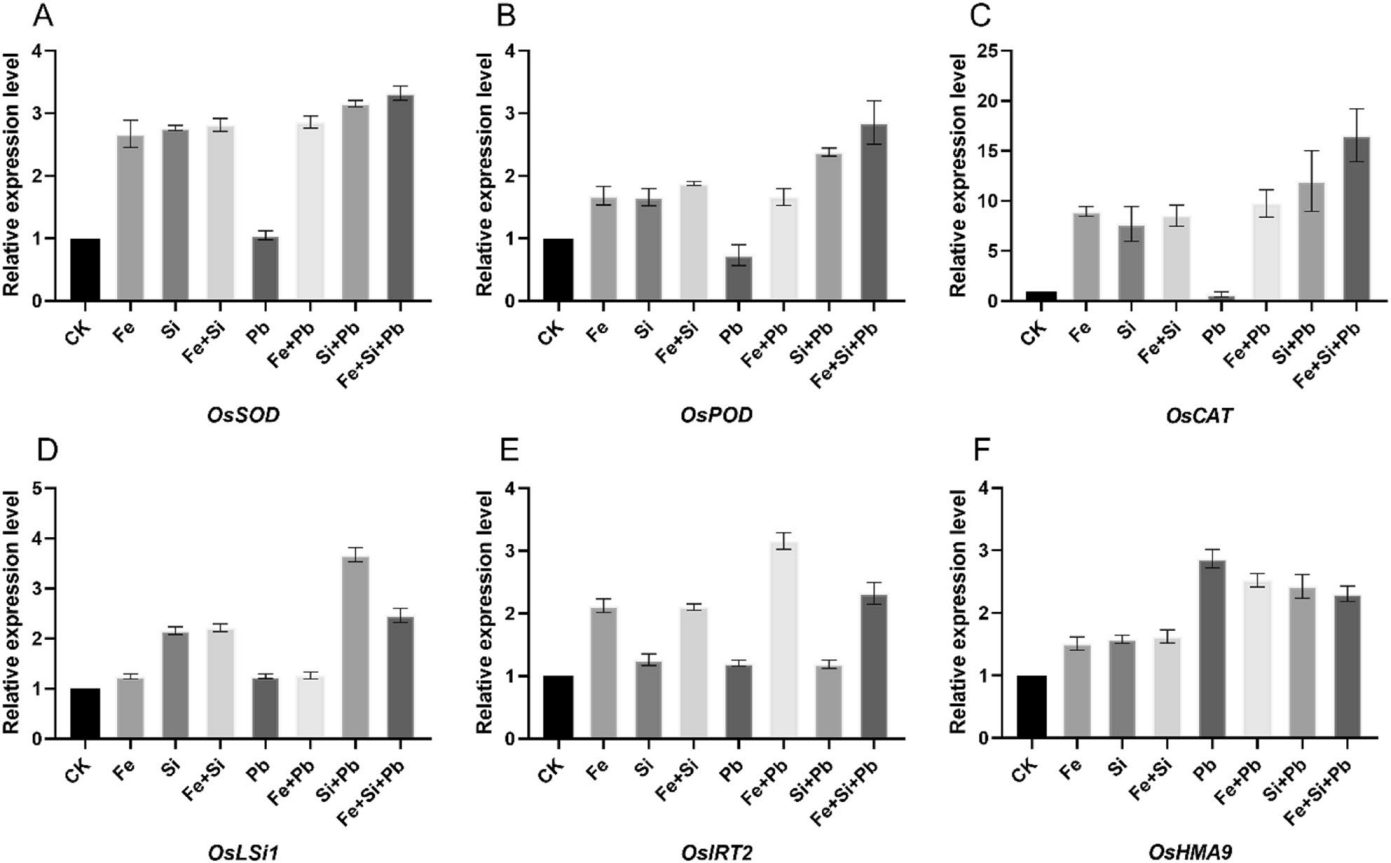
Influence of Pb, Si, and Fe nanoparticles on the degree of metal transporters and antioxidant-related genes' expression. (**A**) superoxide dismutase (SOD), (**B**) peroxidase (POD), (**C**) catalase (CAT), (**D**) *OsLSi1,* (**E**) *OsIRT2*, and (**F**) *OsHMA9* in rice seedlings.

The qRT-PCR measured the relative expression patterns of genes that code for antioxidants. Pb triggered a remarkable decline in the degree of these genes' expression. In contrast, Si and Fe nanoparticle application in the rice seedlings improved these genes' expression patterns under Pb stress (Fig. 5). Moreover, we evaluated the expression patterns of metal transporter genes. For example, *OsHMA9* (metal or Pb transporter) displayed the highest expressions under Pb exposure compared to Pb + Si + Fe treatments. In contrast, the lowest expression levels were detected under the combined or alone application of Fe and Si. *OsLSi1* (a-Si transporter) showed the highest expression pattern under Pb + Si, followed by Pb + Si + Fe treatment, while the lowest was under Fe and Pb treatments. *OsIRT2* (a Fe transporter) expression was higher in treatments with Fe NPs than others, with Pb + Fe treatment having the most significant level followed by Pb + Fe + Si treatment, and Pb and Pb + Si treatment having the lowest level.

### Correlation, heatmap, homogeneity, and principal component analysis

**Figure 6:**
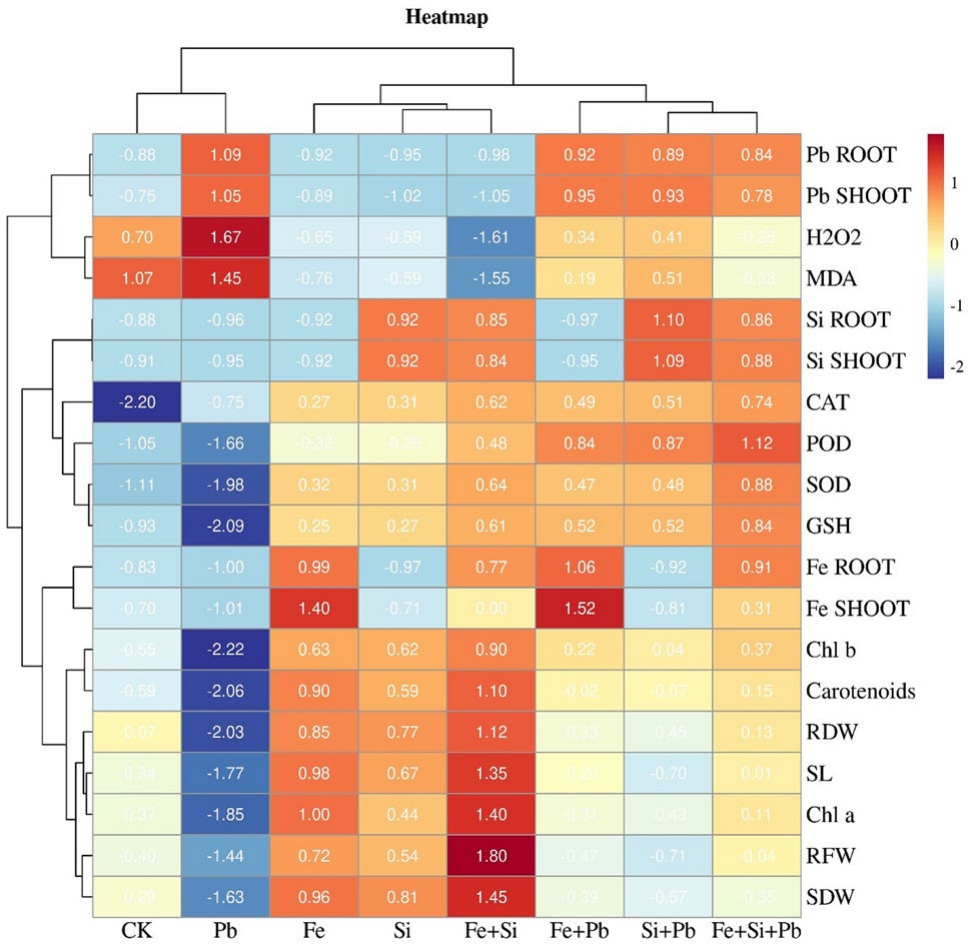
Heat map of morphological and physiological indices of rice treated with silicon, iron, and lead. The indices include Fe nanoparticles contents in roots (Fe Root), shoots (Fe Shoot), Si nanoparticles contents in roots (Si Root), shoots (Si Shoot), Pb contents in roots (Pb Root), shoots (Pb Shoot), malondialdehyde (MDA), superoxide dismutase (SOD), shoot length (SL), chlorophyll a (Chl a), peroxidase (POD), chlorophyll b (Chl b), root fresh weight (RFW), carotenoids (CAR), glutathione (GSH), catalase (CAT), shoot length (SL), hydrogen peroxide (H_2_O_2_), and root length (RL).

According to the grey correlation degree results, the correlation degree among indicators is substantial. All indicators have a correlation degree of more than 0.6 with the content of Pb in the above-ground parts (Fig. S4). The correlation degree of lead content with Fe contents in the shoot, Si contents in the root and shoot, and SOD is high, reaching 0.7. The concentrations of H_2_O_2_ and MDA in rice treated with Pb were the highest, indicating that Pb toxicity in rice was significant. But CAT, SOD, POD, GSH, Chl a, and other physiological indices decreased (Fig. 6). Physiological indices such as Chl a, carotenoids, RDW, SL, RFW, and SDW were higher in plants treated with Fe and Si nanoparticles alone or in combination than Pb treated plants. However, these indicators increased slightly with Fe and Pb than Si and Pb. As a result, in this experiment, Fe has a greater ability to mitigate Pb toxicity than Si. Furthermore, Pb content in root and ground parts was lower when treated with Si + Fe + Pb than when treated with Fe and Pb or Si and Pb alone.

According to the principal component analysis, PCA1 accounted for 49.67%, and PCA2 accounted for 32.31%, a total of 81.98% (Fig. S5). The larger the absolute value of Cohen's d, the larger the effect size (Table 2). In the study, we observed significant and substantial differences (large effect sizes) in morphological traits and antioxidant enzyme activities when Si and Fe nanoparticles were applied. The effect sizes observed for the combined application of both Si and Fe nanoparticles were even larger when compared to the effect sizes observed when each nanoparticle was applied individually (sole application). Regarding PCA1, Pb content in roots was significantly increased, while carotenoids, RFW, SDW, and RDW were decreased considerably. In the dimension of PCA2, H_2_O_2_, GSH, and SOD decreased significantly, and H_2_O_2_ decreased the most. According to the correlation coefficient, most indicators have a significant association. Pb content was negatively associated with the content of Fe and Si, and the content of Si in the root was negatively linked with Fe content in the ground-parts (Table S2). The concentrations of Pb in root and shoot were positively correlated with a correlation coefficient of 0.996. The correlation coefficient between CAT and SOD was 0.953. Moreover, the Shapiro–Wilk Test (Normality test) results indicate that all data conform to a normal distribution (Table S3). The homogeneity of variance of Levene's Test also shows that the variances are comparable (Table S4). There are no large or small outliers in the data of this experiment.

**Table 2:** Effect sizes of Pb, silicon, and iron nanoparticles on various parameters based on Cohen’s criteria.

	Effect size (r)						
	Fe	Si	Pb	Fe × Si	Fe × Pb	Si × Pb	Fe × Si × Pb
SL	0.908	0.899	0.778	0.962	0.124	0.308	0.501
RFW	0.832	0.784	0.816	0.979	0.021	0.429	0.656
RDW	0.811	0.891	0.939	0.952	0.386	0.524	0.209
SDW	0.933	0.822	0.951	0.961	0.201	0.636	0.125
Pb root	0.407	0.649	0.998	0.766	0.999	0.998	0.991
Pb shoot	0.702	0.938	0.996	0.880	0.995	0.999	0.998
Fe root	1.000	0.922	0.835	0.999	0.998	0.704	1.000
Fe shoot	0.998	0.018	0.847	0.981	0.996	0.354	0.987
Si root	0.908	0.999	0.976	0.999	0.976	0.997	0.997
Si shoot	0.032	0.996	0.522	0.998	0.547	0.999	0.995
SOD	0.989	0.983	0.843	0.992	0.994	0.993	0.997
POD	0.820	0.748	0.764	0.958	0.982	0.989	0.990
CAT	0.984	0.9785	0.922	0.998	0.993	0.996	0.998
GSH	0.972	0.969	0.927	0.984	0.976	0.975	0.988
Chl a	0.977	0.923	0.990	0.993	0.029	0.023	0.912
Chl b	0.977	0.879	0.856	0.919	0.716	0.674	0.679
Carotenoids	0.964	0.878	0.976	0.944	0.760	0.786	0.934
H_2_O_2_	0.862	0.862	0.630	0.950	0.184	0.171	0.767
MDA	0.958	0.949	0.728	0.984	0.917	0.757	0.964

Small effectη2 = 0.04; Moderate effectη2 = 0.25; Large effectη2 = 0.64. Please see Table 1 for traits abbreviations.

## Discussion

The current findings supported the hypothesis that the synergistic application of Fe- and Si-NPs effectively mitigated Pb stress in rice. This was achieved through the enhancement of enzymatic activities and the reduction of oxidative stress, ultimately resulting in decreased Pb uptake by the plants. Heavy metal stress, particularly Pb, can cause poisonous and environmental pollution. Pb disturbs the enzyme activities that lead to changes in mineral nutrition and membrane permeability, suppressing plants' growth, photosynthesis, and morphological characteristics^47^. Several impacts on plant growth and development, accompanied by noticeable symptoms, including inhibited root development, smaller leaves, and stunted growth, have already been reported under Pb stress^48^. In maize, Pb reduces the primary root growth and decreases the number of branches in seedlings^49^. A previous study showed that Pb toxicity exhibited heavy metal toxicity symptoms such as a brittle texture, stunting, and blackening of roots, severely disturbing the growth of banana (*Musa acuminata* L.) seedlings^50^. Here, plant growth parameters, including shoot length and biomass accumulation, were suppressed by the excess quantity of Pb in rice seedlings (Fig. 1). The Pb toxicity also inhibited chlorophyll synthesis (Fig. S2) and damaged root ultrastructure. A significantly high content of Pb was found in both roots and shoots of rice. Intriguingly, the introduction of Si- and Fe-NPs had a notable positive impact on various aspects of plant health. Specifically, their application led to a significant enhancement of plant growth parameters, such as shoot length and biomass accumulation. This positive effect on biomass accumulation suggests an overall improvement in the plants' physiological and metabolic activities. Furthermore, the introduction of Si- and Fe–NPs resulted in a substantial reduction in the concentration of Pb detected in both the root and shoot parts of rice plants. This indicates that Si- and Fe–NPs played a crucial role in mitigating the adverse effects of Pb stress. The reduction in Pb content suggests a potential mechanism where Si- and Fe–NPs contribute to limiting the uptake or translocation of Pb within the plant, thus alleviating the negative impacts of Pb on growth and development.

It is well known that Pb prompts phytotoxicity in seedlings and can increase the rate of ROS and oxidative stress, which can change the production of macromolecules, such as nucleic acids, lipids, and proteins in cells^13^. In plant cells, heavy metals triggered by oxidative stress can either repress the production level of antioxidative enzymes or stimulate more ROS^51^. Here, rice seedlings exposed to Pb displayed considerably higher contents of H_2_O_2_ and MDA (lipid peroxidation) than the control, and the high rate of lipid peroxidation indicates more damage to lipids (Fig. 3). However, when rice seedlings were treated with a combination of Si- and Fe-NPs along with Pb, the MDA contents were lower compared to control plants. This suggests that the co-application of Si- and Fe–NPs with Pb has a protective effect, mitigating the metal toxicity and inhibiting cell membrane destruction caused by Pb-induced oxidative stress. These findings align with previous research that demonstrated the positive role of NPs in reducing the toxicity of other heavy metals, such as Cd, in rice. Specifically, earlier studies have shown that NPs can effectively reduce the MDA concentration, indicating a potential mechanism through which NPs contribute to alleviating oxidative stress and associated damage in plant cells exposed to heavy metals^44,52^.

The improved antioxidative defense regulatory enzymatic activities, including CAT, SOD, and POD (Fig. 3), will create tolerance in stressed plant cells against severe oxidative cellular damage generated under Pb toxicity in plants through excessive ROS production. SOD acts as the foremost important defense antioxidative enzyme to salvage the extra ROS by regulating the production of superoxide (O_2_^−^)^53^. POD also plays a crucial role in scavenging the excess ROS in plant tissues under heavy metal toxicity^54^. Pb heavy metal treatment caused declining SOD enzyme activity in cotton (*Gossypium hirsutum* L.) seedlings because of high oxidative stress that might not have been enough to reduce the high accumulation of ROS produced by higher MDA contents than their controls. Here, heavy metal-induced cell toxicity caused an increased accumulation of ROS in rice plants under Pb-treatment, reducing the SOD enzyme activity. In the co-exposure scenario of Si- and Fe–NPs along with Pb heavy metals, the increased SOD enzyme activity in rice seedlings suggests a potential mitigating effect on Pb-induced oxidative stress. This elevation in SOD activity can be attributed to the lower levels of ROS observed in the rice seedlings exposed to Si- and Fe–NPs compared to those treated with Pb alone. The presence of Si- and Fe–NPs seems to contribute to a reduced ROS burden within the plant cells, creating an environment with lower oxidative stress. Consequently, the SOD enzyme, being a crucial defense mechanism against excess ROS, experiences an upregulation in its activity. This increased SOD activity can be seen as a protective response, as it helps the plant cells manage and neutralize the potentially harmful effects of ROS, contributing to an overall enhancement of antioxidative defense mechanisms.

Similarly, positive effects of NPs were detected in other plant species, such as barley^55^, rice^52,56,57^, wheat^58,59^, and okra^60^. Co-application of Si- and Fe-NPs with heavy metals triggered the CAT activity, which was also reduced in Pb-stressed plants. We noticed a substantial rise in the CAT activity in the plants with co-exposure of Si- and Fe–NPs under Pb metal treatments than control (Fig. 3). Both POD and CAT enzymes are crucial in reducing ROS accumulation and improving plant cell defense against heavy metal-induced oxidative stress. POD is a significant scavenging enzyme for H_2_O_2_ under abiotic stress in plant cells. The co-application of Si and Fe-NPs with Pb heavy metal ions resulted in a notable increase in POD activity. This heightened POD activity indicates a reduction in H_2_O_2_ levels within the rice seedlings, showcasing an effective mitigation of oxidative stress induced by Pb exposure. This observation aligns with recent research, such as the study in wheat, which demonstrated that the use of Fe-NPs increased the activity of the POD enzyme under exposure to Cd^39^. The collective presence of Si and Fe-NPs, along with heavy metals, exhibited an enhanced antioxidant activity within the plant tissues compared to seedlings treated solely with Pb. This suggests a synergistic effect between Si and Fe-NPs in alleviating oxidative stress, contributing to improved plant defense mechanisms against the detrimental impacts of heavy metal exposure.

The ROS scavenging systems or GSH can reclaim heavy metals in the vacuole^61,62^. GSH can construct a complex with metals that, by vacuolar compartmentalization, could detoxify the heavy metals^52,61^. Applying Si and Fe-NPs boosted the GSH concentration compared to the Pb treatment alone. Hence, the high activity of GSH helps in the detoxification of Pb. Si and Fe-NPs significantly reduced Pb uptake and translocation to leaves, decreasing Pb toxicity in rice seedlings. We concluded that alleviating oxidative stress in leaves and improving an antioxidant defense mechanism by Si and Fe-NPs may be directly associated with decreased Pb accumulation in leaves. Our results revealed that Fe and Si-NPs enhanced the expression patterns of CAT, SOD, and POD genes in comparison to the control, both alone and in combination (Fig. 5). Pb triggered a remarkable decrease in the degree of these genes' expression, whereas both Si- and Fe–NPs application in the rice seedlings improved the patterns of these genes' expression under Pb stress (Fig. 5). The increased expression of these genes (*OsSOD*, *OsPOD*, and *OsCAT)* were most likely due to the emission of Si and Fe ions from NPs. Importantly, the expression data are consistent with the minimal Pb deposition in rice shoots subjected to Pb + NPs versus Pb treatment.

Several genes are involved in plants' metal and heavy metal metabolism, from transport to bioaccumulation and assimilation^63^. The mechanism was further refined by analyzing the genes involved in Pb absorption and translocation. *OsHMA9*, is identified as a key transporter gene involved in the uptake and bioaccumulation of Pb^64^. Under normal conditions, *OsHMA9* expression is closely linked to the transportation of Pb within plants, indicating its significant role in Pb uptake and transport. Our results revealed that under the sole use of Fe- and Si-NPs treatments, the *OsHMA9* expression recovered to control levels, showing that Pb absorption was successfully blocked. The higher expression levels of *OsHMA9* in response to Pb exposure suggested its involvement in Pb uptake/transport, which was consistent with prior research that linked *OsHMA9* to Pb transportation^64^. However, Fe and Si-NPs significantly reduced the expression patterns of *OsHMA9*, which related to the alleviation of Pb toxicity by NPs. Under Fe-NPs induction, *LCT1* expression increased dramatically in shoots, highlighting the excess of iron transporter proteins. Due to the favorable link between *LCT1* expression and iron ions, it is suggested that the Fe ions emission from Fe-NPs causes elevated expression patterns of *LCT* in the upper part of plants^65^. Additionally, *OsLSi1* is a gene associated with Si transport in rice plants^24,66^. The study reveals that Si-NPs enhanced the expression patterns of *OsLSi1*. This increase in expression is noteworthy for its association with increased antioxidant activity and the transport of Si within rice plants. The application of Si-NPs contributes to the mitigation of Pb toxicity by influencing the expression of *OsLSi1*. Furthermore, *OsIRT2* emerges as a gene linked to Fe absorption in rice roots^67^. Under the influence of Fe-NPs, *OsIRT2* is significantly up-regulated in rice roots. This upregulation is associated with enhanced Fe absorption in the roots, and the study suggests that the increased expression of *OsIRT2* contributes to the alleviation of Pb toxicity. Here, high expression levels of *OsLSi1* and *OsIRT2* were detected under the application of Si and Fe nanoparticles, which also increased antioxidant enzymes activities and reduced ROS. These findings showed that Si- and Fe-NPs inhibited the expression patterns of Pb uptake and transporter genes in rice exposed to Pb stress. A recent study found that *OsLSi1* reduces antioxidant activity and transports Si in rice^68^. Similarly, *OsIRT2* linked to Fe absorption in rice was significantly up-regulated in rice roots under Fe application^24,66,69^. In short, these genes—*OsHMA9*, *OsLSi1*, and *OsIRT2*—play distinct roles in the context of Pb toxicity in rice plants. *OsHMA9* is involved in Pb uptake and transport, *OsLSi1* is associated with Si transport and antioxidant activity, and *OsIRT2* is linked to Fe absorption. The interplay of these genes and their expressions collectively contribute to the effective reduction of Pb bioaccumulation and associated toxicity in rice plants.

We detected high Pb contents in roots and shoots, which may result in cellular structural damage and induce abnormalities (Fig. 4). In the present study, TEM observation displayed severe destruction of rice root cortex cells in Pb-stressed plants. In contrast, Si- and Fe-NP treatments enhanced the integrity of cell structure. A previous study showed that Si could recover cell ultrastructure in Cd-treated maize plants^70^. Here, Pb-treated cells displayed more cell abnormalities, such as reduced root cell size and expanded cortical cell diameter, according to semi thin section analyses (Fig. 4). These findings agreed with prior studies that found comparable anomalies under cadmium exposure^44,71^. Our findings displayed that the co-application of Fe- and Si-NP considerably enhanced the root ultrastructure properties and morphological traits under Pb stress. These cell structural and morphological improvements could be induced by the progressive effect of Si and Fe-NP on heavy metals, which previous investigations have described^28,72,73^. Si can co-precipitate with heavy metals, whereas Si is present in various root cell wall regions^74^. Here, vacuoles and cell walls were damaged by Pb treatment, but Si and Fe-NP treatments relieved cell structure.

**Figure 7:**
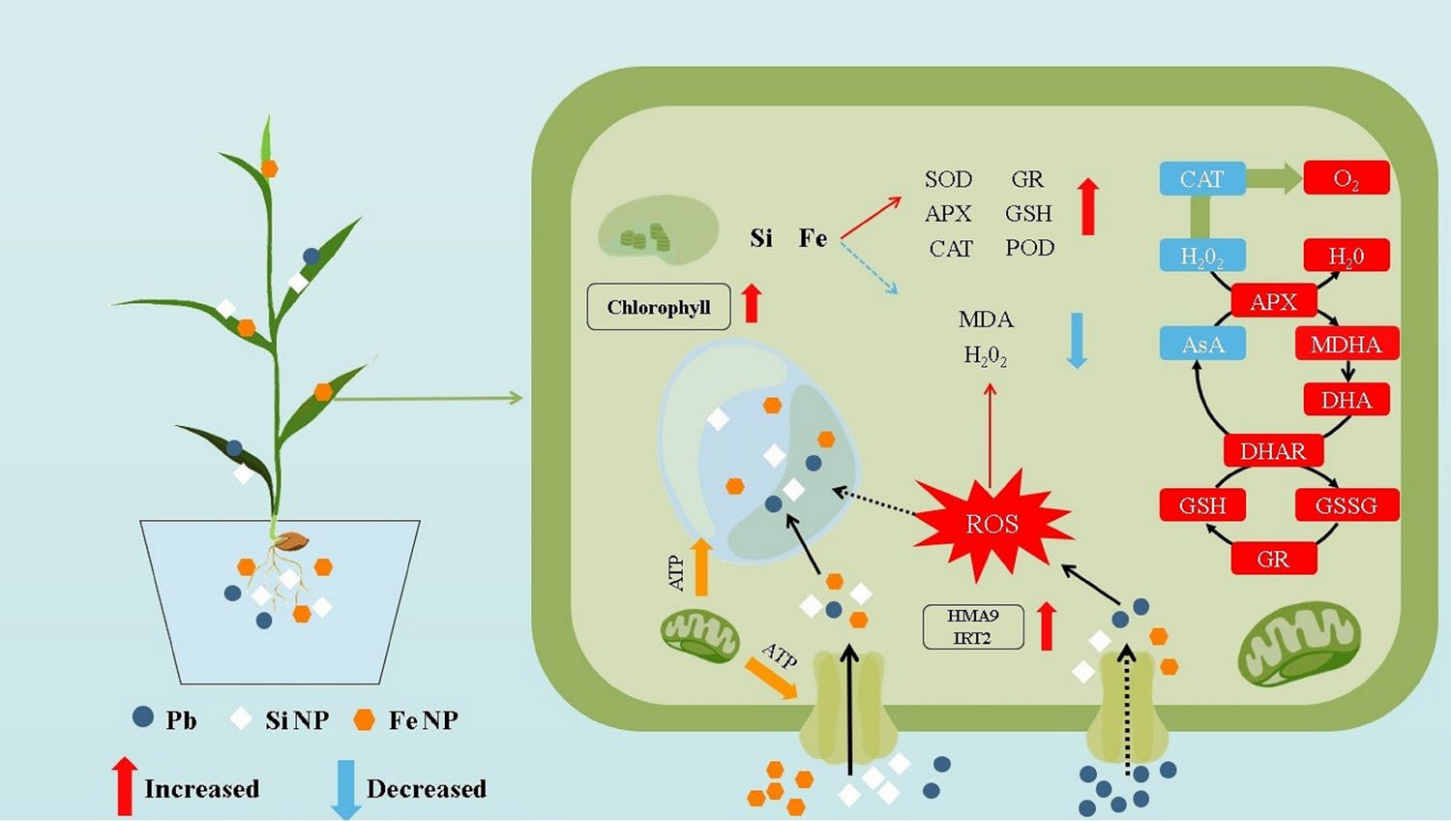
The theorized mechanisms by which iron oxide (Fe_2_O_3_) and silicon oxide (SiO_2_) nanoparticles alleviate lead (Pb) toxicity. CAT, catalase; ROS, reactive oxygen species; POD, peroxidase; SOD, superoxide dismutase; As a result of oxidative stress, destruction of cell membranes, upregulation of metal transporters, impairment of photosynthesis, and plant growth, rice plants subjected to Pb stress have higher concentrations of Pb in their roots and shoots. Application of synergistic exogenous Fe- and Si-NPs reduces Pb toxicity in roots and shoots, boosts photosynthesis and nutritional uptake, and strengthens Fe- and Si-mediated signaling and antioxidative defense. The up-regulation depicted by the red arrow and the down-regulation depicted by the green arrow both occur in response to Fe- and Si-NPs under Pb stress.

In view of these findings, we suggest a scheme to characterize the mechanisms by which Si- and Fe–NPs reduce Pb stress (Fig. 7). Increased nutrient absorption, repair of Pb-induced ultra-structural damages, and activation of rice's antioxidative defense capabilities are all ways in which Si- and Fe–NPs help rice seedlings recover from the growth inhibition caused by Pb toxicity. Fe^2+^ from FeO-NPs and Pb^2+^ competing for the same metal transporters may also limit Pb ion absorption and accumulation in rice seedlings (Fig. 7). While our study provides valuable insights into the potential of Si and Fe-NPs in mitigating Pb toxicity in rice plants, it is important to acknowledge certain limitations. Firstly, the study primarily focused on the short-term effects, and long-term implications of NPs application, which need further investigation to understand their sustained efficacy and any potential ecological impacts. Additionally, the study was conducted under controlled laboratory conditions, and the translation of these findings to real-world field scenarios requires careful consideration. The specific mechanisms of NPs interaction with soil microbes and their long-term effects on soil health and ecosystem stability remain areas of uncertainty. Future research should address these limitations by conducting field trials to validate the effectiveness of Si and Fe nanoparticles in actual contaminated soil environments. Furthermore, assessing the potential risks associated with the application of NPs, such as unintended consequences on non-target organisms and soil microbial communities, should be a focus of future investigations. Recommendations include comprehensive field studies, assessing the long-term effects, and incorporating ecological considerations to ensure the safe and effective application of Si and Fe nanoparticles for mitigating heavy metal toxicity in agricultural soils.

## Conclusions

Our results showed that Pb stress caused severe damage to rice seedlings, which reduces enzymatic activities and biomass accumulation. The modifications in the POD, CAT, and SOD enzymatic activities of leaves displayed the effects of Pb toxicity, which produce free oxygen radicals. TEM and semi-thin sectioning allowed us to identify many cell abnormalities, such as malformed cell walls, mitochondria, pericycle, epidermis, endodermis, and cortex. However, the synergistic use of Fe- and Si-NPs repressed the lethal effects of Pb in rice seedlings by altering the cellular, metabolic, and morphological traits. The results of Cohen's d and PCA analysis indicated a significant and strong positive correlation between the presence of Fe- and Si-NPs with the observed morphological characteristics and antioxidant activities under Pb toxicity. Both Si and Fe-NPs can suppress oxidative stress and adjust the antioxidant defense system in plants by modulating the expression patterns of metal transporters, such as *OsHMA9*, *OsLSi1,* and *OsIRT2*. These results indicate that the combined use of Si- and Fe NPs can mitigate the harmful effects of Pb exposure on rice plants more effectively than using either kind of nanoparticle alone. Si- and Fe-NPs play the most critical role in reducing Pb concentrations in rice and preventing its translocation from the plant's roots to aerial portions, finally reducing cell damage.

### Supplementary Information

Supplementary Information.

## Data Availability

The datasets used and/or analyzed during the current study are available from the corresponding author on reasonable request.
