# Supplementary material for: Silicon and iron nanoparticles protect rice against lead (Pb) stress by improving oxidative tolerance and minimizing Pb uptake
Source: Sci Rep. 2024 Mar 12;14:5986. doi: 10.1038/s41598-024-55810-2 (PMC10933412; doi:10.1038/s41598-024-55810-2)
Supplement: Supplementary file 1 — Supplementary Information. [file 41598_2024_55810_MOESM1_ESM.docx]

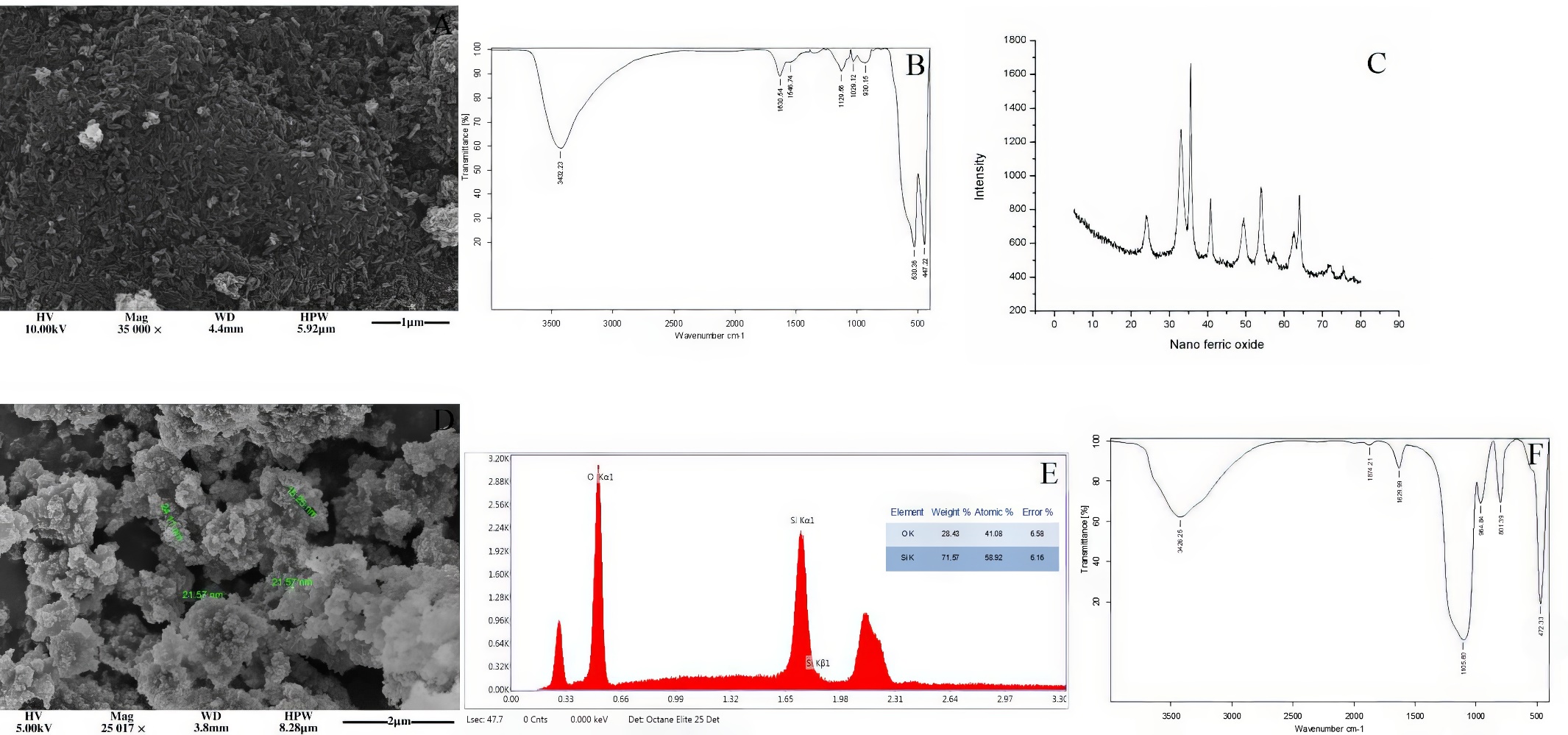


Fig S1: Fe (Fe NPs) and Si (Si NPs) nanoparticles' characterization. A: Scanning electron microscope picture of Fe nanoparticles. B: FTIR spectrum of Fe-NPs. C: XRD pattern of Fe-NPs.

D: Scanning electron microscope picture of Si nanoparticles. E: Scanning electron microscope energy spectrum analysis of Si nanoparticles. F: FTIR spectrum of Si NPs


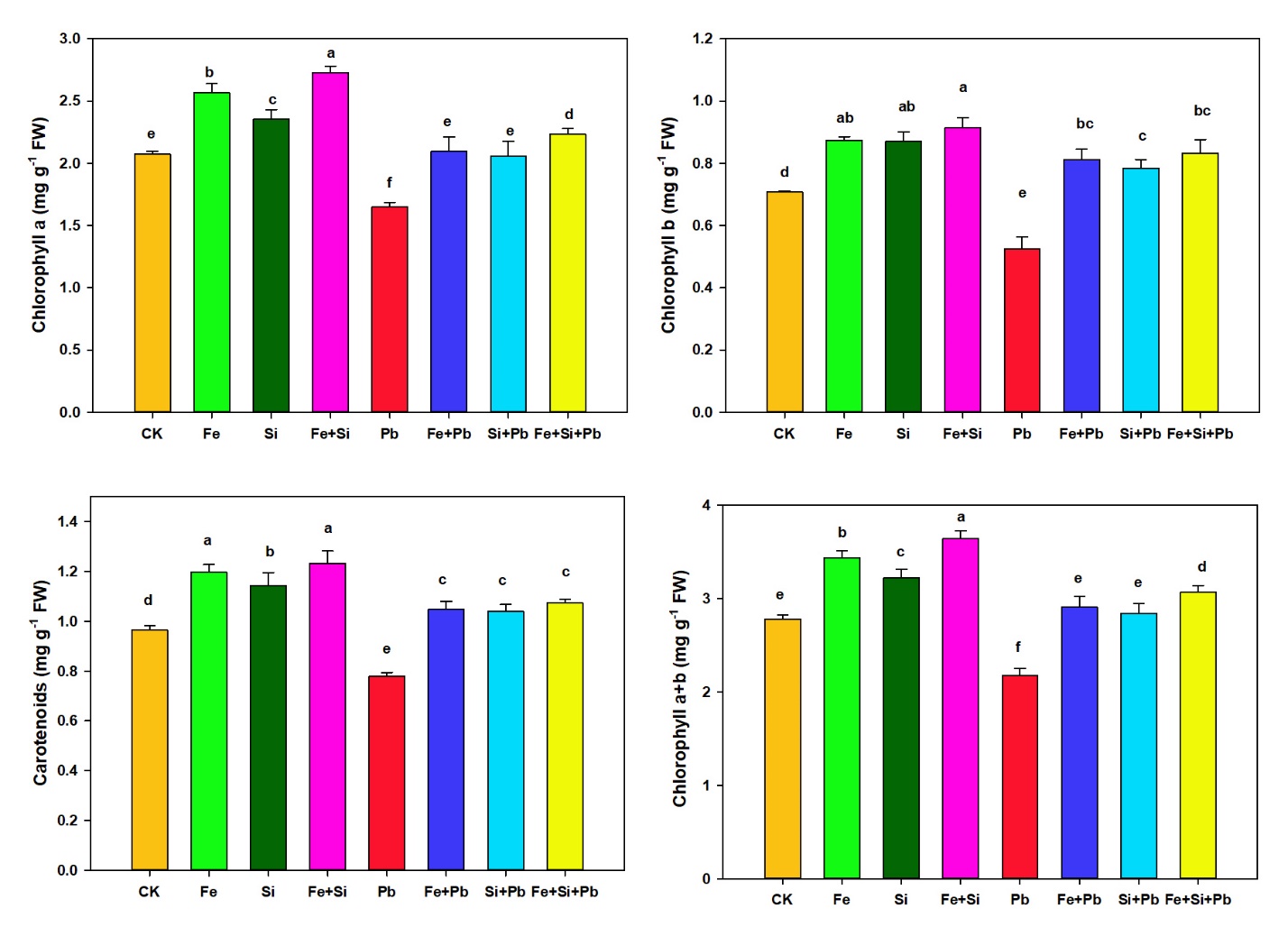


Fig S2: Chlorophyll concentrations under Pb and nanoparticles treatments in rice.

Small letters above the bar show the significant difference, and results are denoted by the LSD test and mean ±SD at p ≤0.05.

**
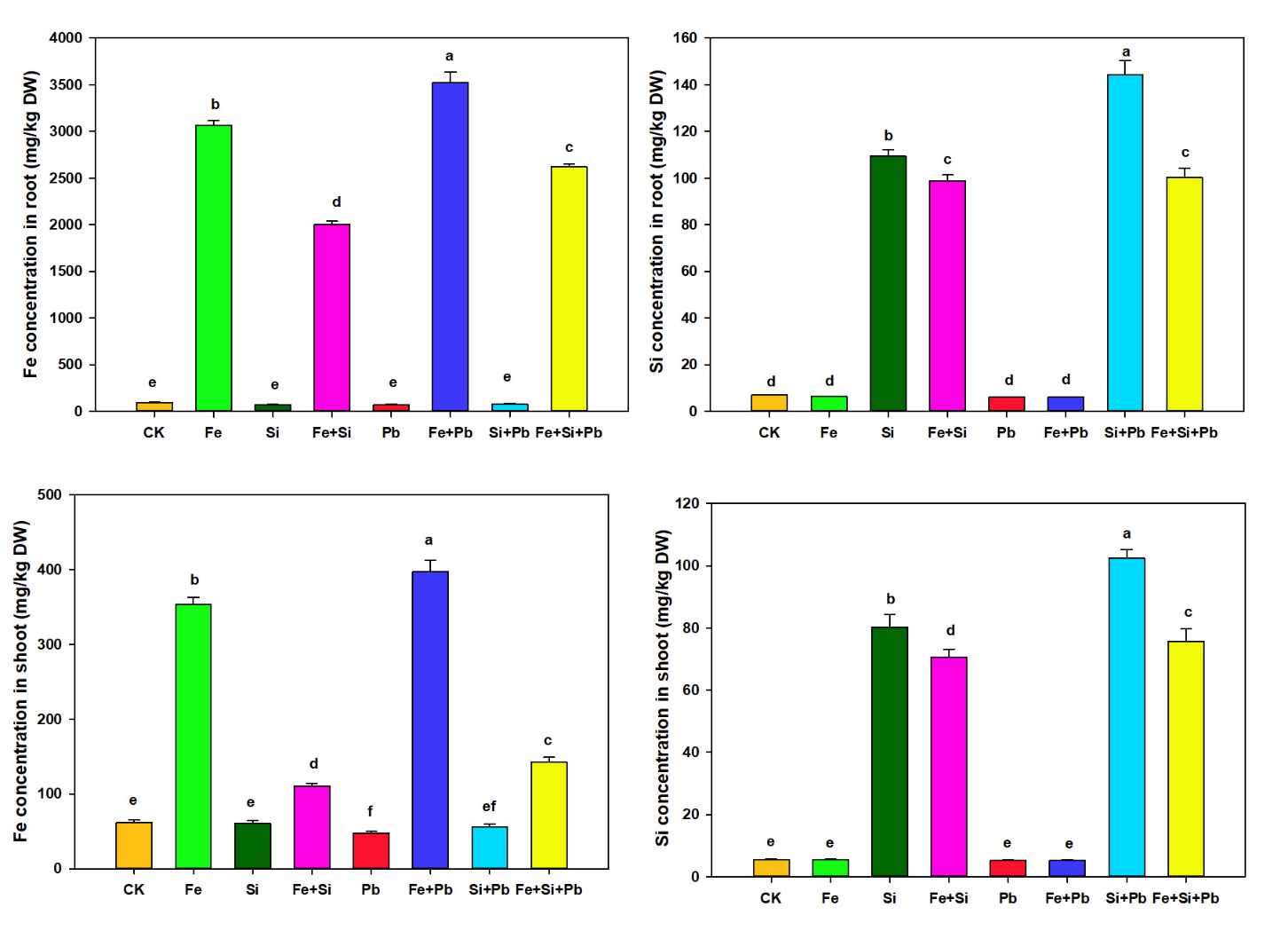
**

Fig S3: Concentrations of Si and Fe in rice plants under Si and Fe nanoparticles.

Small letters above the bar show the significant difference, and results are denoted by the LSD test and mean ±SD at p ≤0.05.


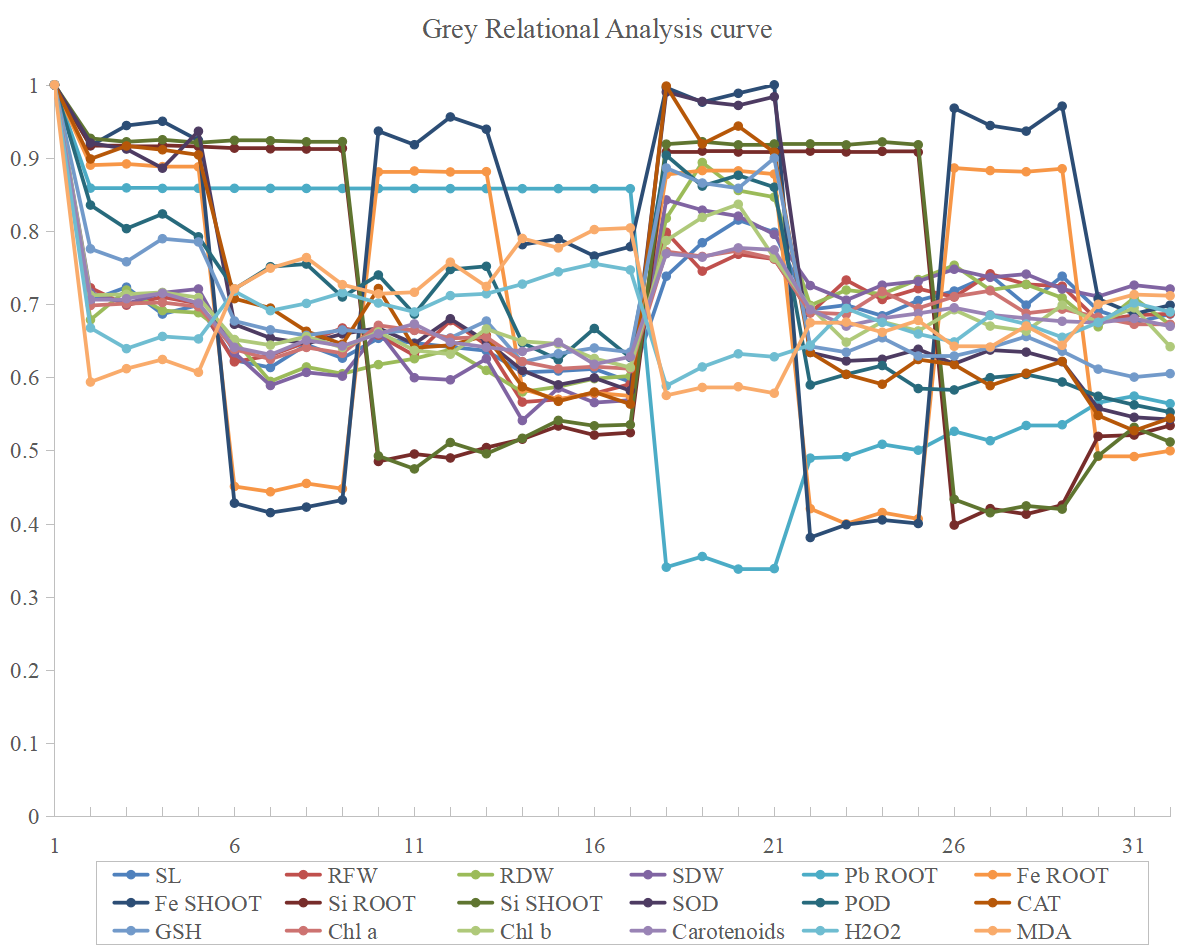
Fig S4: Grey correlation analysis of rice plants treated with Si, Fe-NPs, and Pb.

The indices include Fe nanoparticles contents in roots (Fe Root), shoots (Fe Shoot), Si nanoparticles contents in roots (Si Root), shoots (Si Shoot), Pb contents in roots (Pb Root), shoots (Pb Shoot), malondialdehyde (MDA), superoxide dismutase (SOD), shoot length (SL), chlorophyll a (Chla), Peroxidase (POD), chlorophyll b (Chlb), root fresh weight (RFW), carotenoids (CAR), glutathione (GSH), catalase (CAT), shoot Length (SL), hydrogen peroxide (H_2_O_2_), and root length (RL).


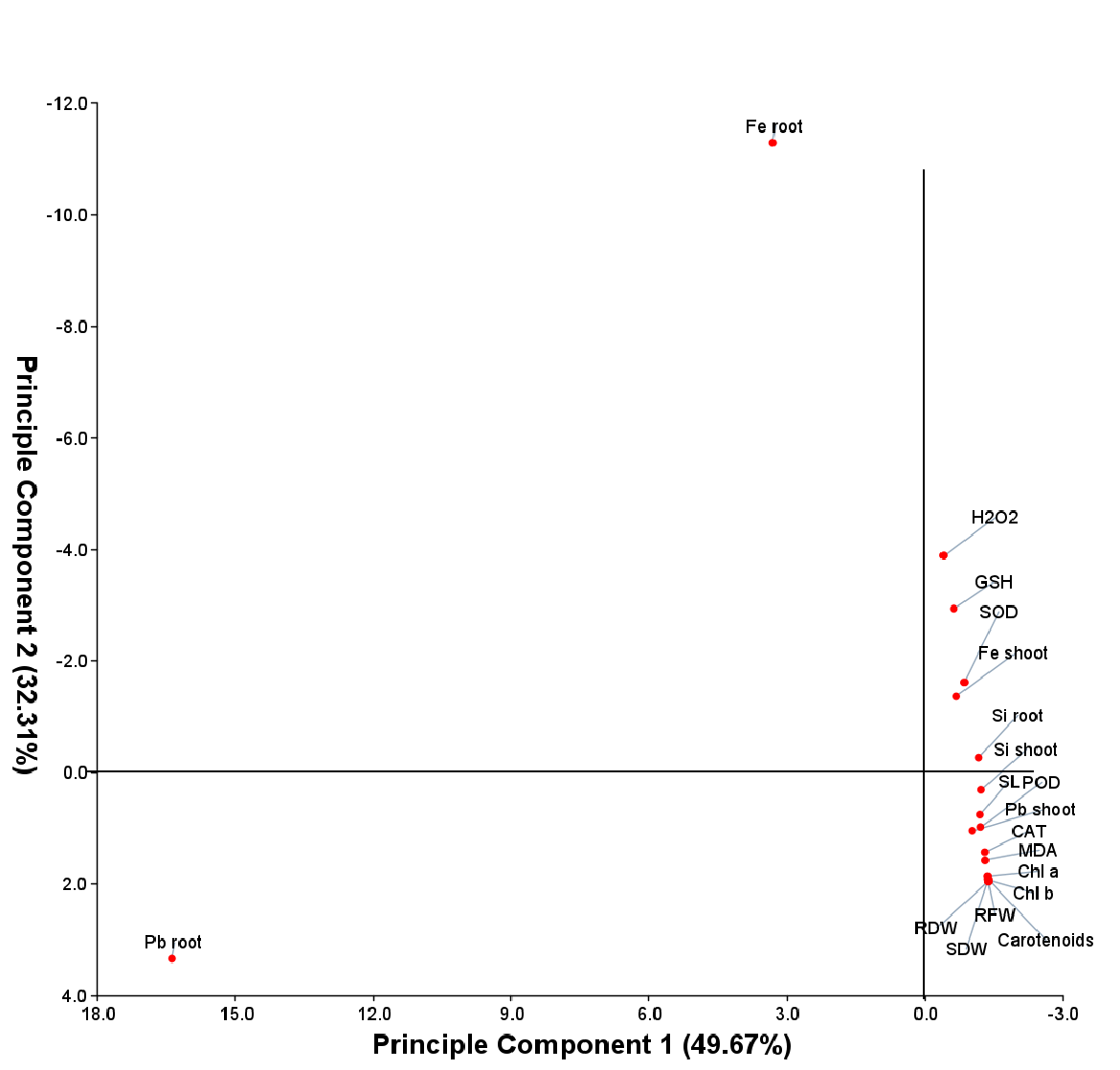


Fig S5: Principal component analysis of morphological and physiological indices of rice treated with silicon, iron and lead.

The indices include Fe nanoparticles contents in roots (Fe Root), shoots (Fe Shoot), Si nanoparticles contents in roots (Si Root), shoots (Si Shoot), Pb contents in roots (Pb Root), shoots (Pb Shoot), malondialdehyde (MDA), superoxide dismutase (SOD), shoot length (SL), chlorophyll a (Chla), Peroxidase (POD), chlorophyll b (Chlb), root fresh weight (RFW), carotenoids (CAR), glutathione (GSH), catalase (CAT), shoot Length (SL), hydrogen per oxide (H_2_O_2_), and root length (RL).

Table S1. Primer sequences used in the current study for qRT-PCR

| *OsSOD*-F | CAATGCAGCCAGAAGGTGGTG |
| --- | --- |
| *OsSOD*-R | ACAAGCCAAACCCAGCCAGA |
| *OsPOD*-F | CAGCTCTCGGCGACGTTCTA |
| *OsPOD*-R | CGAAGCAGTCGTGGAAGTGC |
| *OsCAT*-F | TCGGCATCCCCACCGATTAC |
| *OsCAT* -R | GTCCATCAAGCAGCTGACGC |
| *OsLSi1-F* | TCACCTCCTCAAGAAGGTCGTGTC |
| *OsLSi1-R* | CACGTCATGAACACCAGCAGGAAC |
| *OsIRT2-F* | AATCGCGTCATTGTGCAGGTTC |
| *OsIRT2-R* | GAACATCTGGTGGAAGCACAGC |
| *OsHMA9-F* | TCACAGGTGACAACTGGAGAACCG |
| *OsHMA9-R* | TCTCACGTCCTCAATGCCAACCTC |

Table S2: Relationship between Pb, Si, Fe nanoparticles and morphological and physiological traits of rice.

|  | Carotenoids | CAT | Chl a | Chl b | Fe ROOT | Fe SHOOT | GSH | H_2_O_2_ | MDA | Pb ROOT | Pb SHOOT | POD | RDW | RFW | SDW | Si ROOT | Si SHOOT | SL | SOD |
| --- | --- | --- | --- | --- | --- | --- | --- | --- | --- | --- | --- | --- | --- | --- | --- | --- | --- | --- | --- |
| Carotenoids | 1 | 0.632** | 0.945** | 0.921** | 0.471** | 0.341 | 0.764** | -0.915** | -0.933** | -0.758** | -0.639** | 0.421* | 0.915** | 0.819** | 0.877** | 0.385* | 0.385* | 0.914** | 0.744** |
| CAT |  | 1 | 0.521** | 0.69** | 0.561** | 0.303 | 0.906** | -0.635** | -0.735** | -0.076 | 0.103 | 0.868** | 0.433* | 0.435* | 0.387* | 0.617** | 0.625** | 0.46** | 0.953** |
| Chl a |  |  | 1 | 0.855** | 0.452** | 0.286 | 0.644** | -0.904** | -0.928** | -0.805** | -0.741** | 0.297 | 0.943** | 0.913** | 0.937** | 0.284 | 0.282 | 0.957** | 0.636** |
| Chl b |  |  |  | 1 | 0.481** | 0.339 | 0.811** | -0.867** | -0.882** | -0.681** | -0.531** | 0.547** | 0.837** | 0.692** | 0.777** | 0.427* | 0.43* | 0.83** | 0.792** |
| Fe ROOT |  |  |  |  | 1 | 0.882** | 0.567** | -0.416* | -0.53** | -0.094 | 0.018 | 0.515** | 0.34 | 0.331 | 0.293 | -0.267 | -0.257 | 0.43* | 0.588** |
| Fe SHOOT |  |  |  |  |  | 1 | 0.362* | -0.21 | -0.326 | -0.064 | 0.031 | 0.284 | 0.207 | 0.129 | 0.183 | -0.485** | -0.482** | 0.291 | 0.336 |
| GSH |  |  |  |  |  |  | 1 | -0.702** | -0.78** | -0.358* | -0.118 | 0.879** | 0.593** | 0.502** | 0.486** | 0.589** | 0.593** | 0.569** | 0.976** |
| H_2_O_2_ |  |  |  |  |  |  |  | 1 | 0.908** | 0.712** | 0.632** | -0.394* | -0.865** | -0.825** | -0.847** | -0.411* | -0.412* | -0.86** | -0.706** |
| MDA |  |  |  |  |  |  |  |  | 1 | 0.635** | 0.548** | -0.461** | -0.874** | -0.853** | -0.869** | -0.412* | -0.413* | -0.903** | -0.794** |
| Pb ROOT |  |  |  |  |  |  |  |  |  | 1 | 0.95** | 0.03 | -0.872** | -0.73** | -0.811** | -0.15 | -0.149 | -0.81** | -0.291 |
| Pb SHOOT |  |  |  |  |  |  |  |  |  |  | 1 | 0.291 | -0.811** | -0.725** | -0.811** | -0.016 | -0.015 | -0.769** | -0.073 |
| POD |  |  |  |  |  |  |  |  |  |  |  | 1 | 0.21 | 0.19 | 0.093 | 0.56** | 0.566** | 0.206 | 0.882** |
| RDW |  |  |  |  |  |  |  |  |  |  |  |  | 1 | 0.872** | 0.928** | 0.296 | 0.304 | 0.942** | 0.576** |
| RFW |  |  |  |  |  |  |  |  |  |  |  |  |  | 1 | 0.921** | 0.282 | 0.282 | 0.902** | 0.515** |
| SDW |  |  |  |  |  |  |  |  |  |  |  |  |  |  | 1 | 0.239 | 0.239 | 0.927** | 0.465** |
| Si ROOT |  |  |  |  |  |  |  |  |  |  |  |  |  |  |  | 1 | 0.996** | 0.224 | 0.605** |
| Si SHOOT |  |  |  |  |  |  |  |  |  |  |  |  |  |  |  |  | 1 | 0.22 | 0.613** |
| SL |  |  |  |  |  |  |  |  |  |  |  |  |  |  |  |  |  | 1 | 0.575** |
| SOD |  |  |  |  |  |  |  |  |  |  |  |  |  |  |  |  |  |  | 1 |

Note: Relationship between rice's morphological and physiological indices and Pb concentration. The correlation coefficient, rho>0.5 and rho<0.5, was calculated by Pearson. The symbols * and ** in the table denote correlations at *P ≤ 0.05* (significant) and *P ≤ 0.01* (highly significant), respectively.

The indices include Fe nanoparticles contents in roots (Fe Root), shoots (Fe Shoot), Si nanoparticles contents in roots (Si Root), shoots (Si Shoot), Pb contents in roots (Pb Root), shoots (Pb Shoot), malondialdehyde (MDA), superoxide dismutase (SOD), shoot length (SL), chlorophyll a (Chla), Peroxidase (POD), chlorophyll b (Chlb), root fresh weight (RFW), carotenoids (CAR), glutathione (GSH), catalase (CAT), shoot Length (SL), hydrogen per oxide (H_2_O_2_), and root length (RL).

Table S3: Testing Normality of all data.

| Testing Normality | | | | | | | |
| --- | --- | --- | --- | --- | --- | --- | --- |
|  |  | Kolmogorov-Smirnov Test |  |  | Shapiro-Wilk Test |  |  |
|  |  | Statistics | df | sig. | Statistics | df | sig. |
| SL | CK | 0.201 | 4 | . | 0.986 | 4 | 0.939 |
|  | Fe | 0.265 | 4 | . | 0.949 | 4 | 0.71 |
|  | Si | 0.156 | 4 | . | 0.994 | 4 | 0.977 |
|  | Fe+Si | 0.332 | 4 | . | 0.843 | 4 | 0.204 |
|  | Pb | 0.264 | 4 | . | 0.918 | 4 | 0.526 |
|  | Fe+Pb | 0.16 | 4 | . | 0.991 | 4 | 0.964 |
|  | Si+Pb | 0.274 | 4 | . | 0.882 | 4 | 0.349 |
|  | Fe+Si+Pb | 0.221 | 4 | . | 0.948 | 4 | 0.702 |
| RFW | CK | 0.244 | 4 | . | 0.911 | 4 | 0.488 |
|  | Fe | 0.214 | 4 | . | 0.963 | 4 | 0.798 |
|  | Si | 0.147 | 4 | . | 0.996 | 4 | 0.985 |
|  | Fe+Si | 0.22 | 4 | . | 0.937 | 4 | 0.637 |
|  | Pb | 0.244 | 4 | . | 0.963 | 4 | 0.799 |
|  | Fe+Pb | 0.183 | 4 | . | 0.981 | 4 | 0.905 |
|  | Si+Pb | 0.223 | 4 | . | 0.973 | 4 | 0.859 |
|  | Fe+Si+Pb | 0.207 | 4 | . | 0.984 | 4 | 0.925 |
| SFW | CK | 0.203 | 4 | . | 0.98 | 4 | 0.899 |
|  | Fe | 0.263 | 4 | . | 0.909 | 4 | 0.479 |
|  | Si | 0.242 | 4 | . | 0.936 | 4 | 0.629 |
|  | Fe+Si | 0.215 | 4 | . | 0.945 | 4 | 0.688 |
|  | Pb | 0.262 | 4 | . | 0.936 | 4 | 0.631 |
|  | Fe+Pb | 0.288 | 4 | . | 0.887 | 4 | 0.369 |
|  | Si+Pb | 0.281 | 4 | . | 0.88 | 4 | 0.337 |
|  | Fe+Si+Pb | 0.331 | 4 | . | 0.865 | 4 | 0.279 |
| BY | CK | 0.407 | 4 | . | 0.702 | 4 | 0.012 |
|  | Fe | 0.266 | 4 | . | 0.879 | 4 | 0.336 |
|  | Si | 0.392 | 4 | . | 0.73 | 4 | 0.024 |
|  | Fe+Si | 0.343 | 4 | . | 0.769 | 4 | 0.057 |
|  | Pb | 0.263 | 4 | . | 0.834 | 4 | 0.179 |
|  | Fe+Pb | 0.214 | 4 | . | 0.954 | 4 | 0.743 |
|  | Si+Pb | 0.195 | 4 | . | 0.973 | 4 | 0.859 |
|  | Fe+Si+Pb | 0.25 | 4 | . | 0.919 | 4 | 0.529 |
| RDW | CK | 0.314 | 4 | . | 0.894 | 4 | 0.4 |
|  | Fe | 0.27 | 4 | . | 0.914 | 4 | 0.503 |
|  | Si | 0.162 | 4 | . | 0.989 | 4 | 0.952 |
|  | Fe+Si | 0.237 | 4 | . | 0.939 | 4 | 0.65 |
|  | Pb | 0.211 | 4 | . | 0.983 | 4 | 0.92 |
|  | Fe+Pb | 0.192 | 4 | . | 0.991 | 4 | 0.961 |
|  | Si+Pb | 0.244 | 4 | . | 0.947 | 4 | 0.7 |
|  | Fe+Si+Pb | 0.345 | 4 | . | 0.846 | 4 | 0.213 |
| SDW | CK | 0.364 | 4 | . | 0.84 | 4 | 0.195 |
|  | Fe | 0.262 | 4 | . | 0.948 | 4 | 0.703 |
|  | Si | 0.261 | 4 | . | 0.885 | 4 | 0.359 |
|  | Fe+Si | 0.267 | 4 | . | 0.951 | 4 | 0.722 |
|  | Pb | 0.23 | 4 | . | 0.964 | 4 | 0.807 |
|  | Fe+Pb | 0.37 | 4 | . | 0.816 | 4 | 0.135 |
|  | Si+Pb | 0.259 | 4 | . | 0.935 | 4 | 0.623 |
|  | Fe+Si+Pb | 0.151 | 4 | . | 0.993 | 4 | 0.972 |
| TDM | CK | 0.214 | 4 | . | 0.981 | 4 | 0.91 |
|  | Fe | 0.265 | 4 | . | 0.938 | 4 | 0.639 |
|  | Si | 0.264 | 4 | . | 0.845 | 4 | 0.21 |
|  | Fe+Si | 0.318 | 4 | . | 0.873 | 4 | 0.31 |
|  | Pb | 0.256 | 4 | . | 0.959 | 4 | 0.774 |
|  | Fe+Pb | 0.165 | 4 | . | 0.99 | 4 | 0.959 |
|  | Si+Pb | 0.183 | 4 | . | 0.993 | 4 | 0.974 |
|  | Fe+Si+Pb | 0.259 | 4 | . | 0.826 | 4 | 0.157 |
| Pb Concentration in root | CK | 0.188 | 4 | . | 0.971 | 4 | 0.845 |
|  | Fe | 0.146 | 4 | . | 0.996 | 4 | 0.986 |
|  | Si | 0.211 | 4 | . | 0.962 | 4 | 0.79 |
|  | Fe+Si | 0.255 | 4 | . | 0.921 | 4 | 0.544 |
|  | Pb | 0.368 | 4 | . | 0.748 | 4 | 0.037 |
|  | Fe+Pb | 0.252 | 4 | . | 0.922 | 4 | 0.551 |
|  | Si+Pb | 0.253 | 4 | . | 0.87 | 4 | 0.299 |
|  | Fe+Si+Pb | 0.334 | 4 | . | 0.777 | 4 | 0.068 |
| Pb Concentration in shoot | CK | 0.152 | 4 | . | 0.997 | 4 | 0.989 |
|  | Fe | 0.189 | 4 | . | 0.991 | 4 | 0.961 |
|  | Si | 0.171 | 4 | . | 0.989 | 4 | 0.952 |
|  | Fe+Si | 0.283 | 4 | . | 0.807 | 4 | 0.116 |
|  | Pb | 0.252 | 4 | . | 0.892 | 4 | 0.395 |
|  | Fe+Pb | 0.182 | 4 | . | 0.974 | 4 | 0.867 |
|  | Si+Pb | 0.232 | 4 | . | 0.971 | 4 | 0.848 |
|  | Fe+Si+Pb | 0.276 | 4 | . | 0.889 | 4 | 0.378 |
| Fe Concentration in root | CK | 0.263 | 4 | . | 0.908 | 4 | 0.474 |
|  | Fe | 0.134 | 4 | . | 0.999 | 4 | 0.997 |
|  | Si | 0.273 | 4 | . | 0.843 | 4 | 0.205 |
|  | Fe+Si | 0.141 | 4 | . | 0.997 | 4 | 0.99 |
|  | Pb | 0.288 | 4 | . | 0.811 | 4 | 0.123 |
|  | Fe+Pb | 0.2 | 4 | . | 0.972 | 4 | 0.851 |
|  | Si+Pb | 0.227 | 4 | . | 0.946 | 4 | 0.694 |
|  | Fe+Si+Pb | 0.287 | 4 | . | 0.868 | 4 | 0.288 |
| Fe Concentration in shoot | CK | 0.247 | 4 | . | 0.907 | 4 | 0.464 |
|  | Fe | 0.184 | 4 | . | 0.977 | 4 | 0.882 |
|  | Si | 0.233 | 4 | . | 0.969 | 4 | 0.837 |
|  | Fe+Si | 0.245 | 4 | . | 0.964 | 4 | 0.801 |
|  | Pb | 0.214 | 4 | . | 0.941 | 4 | 0.66 |
|  | Fe+Pb | 0.344 | 4 | . | 0.845 | 4 | 0.209 |
|  | Si+Pb | 0.279 | 4 | . | 0.864 | 4 | 0.276 |
|  | Fe+Si+Pb | 0.138 | 4 | . | 0.998 | 4 | 0.993 |
| Si Concentration in root | CK | 0.251 | 4 | . | 0.913 | 4 | 0.5 |
|  | Fe | 0.223 | 4 | . | 0.935 | 4 | 0.625 |
|  | Si | 0.173 | 4 | . | 0.981 | 4 | 0.906 |
|  | Fe+Si | 0.203 | 4 | . | 0.983 | 4 | 0.919 |
|  | Pb | 0.278 | 4 | . | 0.878 | 4 | 0.332 |
|  | Fe+Pb | 0.236 | 4 | . | 0.911 | 4 | 0.488 |
|  | Si+Pb | 0.216 | 4 | . | 0.933 | 4 | 0.609 |
|  | Fe+Si+Pb | 0.246 | 4 | . | 0.964 | 4 | 0.802 |
| Si Concentration in shoot | CK | 0.222 | 4 | . | 0.952 | 4 | 0.726 |
|  | Fe | 0.257 | 4 | . | 0.914 | 4 | 0.504 |
|  | Si | 0.235 | 4 | . | 0.971 | 4 | 0.847 |
|  | Fe+Si | 0.332 | 4 | . | 0.871 | 4 | 0.302 |
|  | Pb | 0.34 | 4 | . | 0.859 | 4 | 0.255 |
|  | Fe+Pb | 0.256 | 4 | . | 0.847 | 4 | 0.217 |
|  | Si+Pb | 0.182 | 4 | . | 0.982 | 4 | 0.914 |
|  | Fe+Si+Pb | 0.226 | 4 | . | 0.973 | 4 | 0.863 |
| SOD | CK | 0.227 | 4 | . | 0.97 | 4 | 0.841 |
|  | Fe | 0.192 | 4 | . | 0.984 | 4 | 0.922 |
|  | Si | 0.261 | 4 | . | 0.912 | 4 | 0.494 |
|  | Fe+Si | 0.168 | 4 | . | 0.989 | 4 | 0.955 |
|  | Pb | 0.346 | 4 | . | 0.829 | 4 | 0.164 |
|  | Fe+Pb | 0.251 | 4 | . | 0.914 | 4 | 0.504 |
|  | Si+Pb | 0.26 | 4 | . | 0.867 | 4 | 0.287 |
|  | Fe+Si+Pb | 0.201 | 4 | . | 0.962 | 4 | 0.791 |
| POD | CK | 0.197 | 4 | . | 0.962 | 4 | 0.79 |
|  | Fe | 0.279 | 4 | . | 0.87 | 4 | 0.297 |
|  | Si | 0.367 | 4 | . | 0.761 | 4 | 0.048 |
|  | Fe+Si | 0.263 | 4 | . | 0.907 | 4 | 0.465 |
|  | Pb | 0.254 | 4 | . | 0.862 | 4 | 0.268 |
|  | Fe+Pb | 0.234 | 4 | . | 0.945 | 4 | 0.684 |
|  | Si+Pb | 0.198 | 4 | . | 0.953 | 4 | 0.733 |
|  | Fe+Si+Pb | 0.285 | 4 | . | 0.867 | 4 | 0.286 |
| CAT | CK | 0.178 | 4 | . | 0.981 | 4 | 0.91 |
|  | Fe | 0.23 | 4 | . | 0.943 | 4 | 0.673 |
|  | Si | 0.327 | 4 | . | 0.795 | 4 | 0.094 |
|  | Fe+Si | 0.241 | 4 | . | 0.93 | 4 | 0.593 |
|  | Pb | 0.223 | 4 | . | 0.915 | 4 | 0.51 |
|  | Fe+Pb | 0.218 | 4 | . | 0.956 | 4 | 0.755 |
|  | Si+Pb | 0.23 | 4 | . | 0.925 | 4 | 0.566 |
|  | Fe+Si+Pb | 0.388 | 4 | . | 0.781 | 4 | 0.073 |
| GSH | CK | 0.218 | 4 | . | 0.918 | 4 | 0.523 |
|  | Fe | 0.301 | 4 | . | 0.925 | 4 | 0.563 |
|  | Si | 0.232 | 4 | . | 0.968 | 4 | 0.832 |
|  | Fe+Si | 0.272 | 4 | . | 0.939 | 4 | 0.648 |
|  | Pb | 0.233 | 4 | . | 0.942 | 4 | 0.667 |
|  | Fe+Pb | 0.196 | 4 | . | 0.968 | 4 | 0.828 |
|  | Si+Pb | 0.235 | 4 | . | 0.954 | 4 | 0.739 |
|  | Fe+Si+Pb | 0.179 | 4 | . | 0.985 | 4 | 0.93 |
| Chl a | CK | 0.205 | 4 | . | 0.961 | 4 | 0.786 |
|  | Fe | 0.218 | 4 | . | 0.939 | 4 | 0.647 |
|  | Si | 0.258 | 4 | . | 0.917 | 4 | 0.518 |
|  | Fe+Si | 0.272 | 4 | . | 0.818 | 4 | 0.139 |
|  | Pb | 0.258 | 4 | . | 0.905 | 4 | 0.456 |
|  | Fe+Pb | 0.308 | 4 | . | 0.829 | 4 | 0.165 |
|  | Si+Pb | 0.235 | 4 | . | 0.926 | 4 | 0.572 |
|  | Fe+Si+Pb | 0.301 | 4 | . | 0.832 | 4 | 0.174 |
| Chl b | CK | 0.174 | 4 | . | 0.995 | 4 | 0.983 |
|  | Fe | 0.218 | 4 | . | 0.946 | 4 | 0.692 |
|  | Si | 0.272 | 4 | . | 0.853 | 4 | 0.235 |
|  | Fe+Si | 0.265 | 4 | . | 0.906 | 4 | 0.464 |
|  | Pb | 0.207 | 4 | . | 0.967 | 4 | 0.823 |
|  | Fe+Pb | 0.143 | 4 | . | 0.997 | 4 | 0.991 |
|  | Si+Pb | 0.248 | 4 | . | 0.899 | 4 | 0.427 |
|  | Fe+Si+Pb | 0.294 | 4 | . | 0.857 | 4 | 0.251 |
| Chl a + b | CK | 0.314 | 4 | . | 0.894 | 4 | 0.4 |
|  | Fe | 0.27 | 4 | . | 0.914 | 4 | 0.503 |
|  | Si | 0.162 | 4 | . | 0.989 | 4 | 0.952 |
|  | Fe+Si | 0.237 | 4 | . | 0.939 | 4 | 0.65 |
|  | Pb | 0.441 | 4 | . | 0.63 | 4 | 0.001 |
|  | Fe+Pb | 0.192 | 4 | . | 0.991 | 4 | 0.961 |
|  | Si+Pb | 0.244 | 4 | . | 0.947 | 4 | 0.7 |
|  | Fe+Si+Pb | 0.345 | 4 | . | 0.846 | 4 | 0.213 |
| Carotenoids | CK | 0.249 | 4 | . | 0.963 | 4 | 0.8 |
|  | Fe | 0.236 | 4 | . | 0.971 | 4 | 0.85 |
|  | Si | 0.201 | 4 | . | 0.972 | 4 | 0.852 |
|  | Fe+Si | 0.147 | 4 | . | 0.999 | 4 | 0.996 |
|  | Pb | 0.213 | 4 | . | 0.964 | 4 | 0.802 |
|  | Fe+Pb | 0.188 | 4 | . | 0.966 | 4 | 0.819 |
|  | Si+Pb | 0.2 | 4 | . | 0.961 | 4 | 0.787 |
|  | Fe+Si+Pb | 0.265 | 4 | . | 0.944 | 4 | 0.677 |
| a: Rielly's significance correction | | | | | | | |

Note: Please see Table S2 for traits abbreviations

Table S4: Testing Homogeneity of Variance of all data.

| Testing Homogeneity of Variance | | Levene's Test | df1 | df2 | sig. |
| --- | --- | --- | --- | --- | --- |
| SL | Mean based | 1.116 | 7 | 24 | 0.385 |
|  | Median based | 0.856 | 7 | 24 | 0.554 |
|  | Based on median and with adjusted freedom | 0.856 | 7 | 11.004 | 0.567 |
|  | Based on the clipped average | 1.023 | 7 | 24 | 0.441 |
| RFW | Mean based | 1.25 | 7 | 24 | 0.316 |
|  | Median based | 1.16 | 7 | 24 | 0.361 |
|  | Based on median and with adjusted freedom | 1.16 | 7 | 19.407 | 0.369 |
|  | Based on the clipped average | 1.248 | 7 | 24 | 0.316 |
| SFW | Mean based | 0.898 | 7 | 24 | 0.524 |
|  | Median based | 0.81 | 7 | 24 | 0.588 |
|  | Based on median and with adjusted freedom | 0.81 | 7 | 20.216 | 0.589 |
|  | Based on the clipped average | 0.898 | 7 | 24 | 0.524 |
| BY | Mean based | 1.88 | 7 | 24 | 0.118 |
|  | Median based | 0.769 | 7 | 24 | 0.619 |
|  | Based on median and with adjusted freedom | 0.769 | 7 | 10.612 | 0.625 |
|  | Based on the clipped average | 1.678 | 7 | 24 | 0.162 |
| RDW | Mean based | 0.461 | 7 | 24 | 0.853 |
|  | Median based | 0.282 | 7 | 24 | 0.955 |
|  | Based on median and with adjusted freedom | 0.282 | 7 | 14.907 | 0.951 |
|  | Based on the clipped average | 0.404 | 7 | 24 | 0.89 |
| SDW | Mean based | 2.084 | 7 | 24 | 0.085 |
|  | Median based | 1.691 | 7 | 24 | 0.159 |
|  | Based on median and with adjusted freedom | 1.691 | 7 | 12.647 | 0.198 |
|  | Based on the clipped average | 2.026 | 7 | 24 | 0.093 |
| TDM | Mean based | 1.435 | 7 | 24 | 0.238 |
|  | Median based | 0.885 | 7 | 24 | 0.533 |
|  | Based on median and with adjusted freedom | 0.885 | 7 | 12.838 | 0.544 |
|  | Based on the clipped average | 1.27 | 7 | 24 | 0.306 |
| Pb Concentration in root | Mean based | 5.23 | 7 | 24 | 0.001 |
|  | Median based | 1.552 | 7 | 24 | 0.198 |
|  | Based on median and with adjusted freedom | 1.552 | 7 | 7.265 | 0.284 |
|  | Based on the clipped average | 4.319 | 7 | 24 | 0.003 |
| Pb Concentration in shoot | Mean based | 6.65 | 7 | 24 | 0 |
|  | Median based | 6.124 | 7 | 24 | 0 |
|  | Based on median and with adjusted freedom | 6.124 | 7 | 8.479 | 0.009 |
|  | Based on the clipped average | 6.643 | 7 | 24 | 0 |
| Fe Concentration in root | Mean based | 9.834 | 7 | 24 | 0 |
|  | Median based | 9.587 | 7 | 24 | 0 |
|  | Based on median and with adjusted freedom | 9.587 | 7 | 6.243 | 0.006 |
|  | Based on the clipped average | 9.833 | 7 | 24 | 0 |
| Fe Concentration in shoot | Mean based | 2.702 | 7 | 24 | 0.032 |
|  | Median based | 1.08 | 7 | 24 | 0.406 |
|  | Based on median and with adjusted freedom | 1.08 | 7 | 5.034 | 0.483 |
|  | Based on the clipped average | 2.394 | 7 | 24 | 0.052 |
| Si Concentration in root | Mean based | 3.822 | 7 | 24 | 0.006 |
|  | Median based | 3.39 | 7 | 24 | 0.012 |
|  | Based on median and with adjusted freedom | 3.39 | 7 | 7.578 | 0.058 |
|  | Based on the clipped average | 3.816 | 7 | 24 | 0.006 |
| Si Concentration in shoot | Mean based | 2.574 | 7 | 24 | 0.04 |
|  | Median based | 2.267 | 7 | 24 | 0.064 |
|  | Based on median and with adjusted freedom | 2.267 | 7 | 10.398 | 0.113 |
|  | Based on the clipped average | 2.538 | 7 | 24 | 0.042 |
| SOD | Mean based | 0.661 | 7 | 24 | 0.702 |
|  | Median based | 0.421 | 7 | 24 | 0.879 |
|  | Based on median and with adjusted freedom | 0.421 | 7 | 11.924 | 0.871 |
|  | Based on the clipped average | 0.621 | 7 | 24 | 0.733 |
| POD | Mean based | 1.18 | 7 | 24 | 0.351 |
|  | Median based | 0.42 | 7 | 24 | 0.88 |
|  | Based on median and with adjusted freedom | 0.42 | 7 | 7.457 | 0.864 |
|  | Based on the clipped average | 1.027 | 7 | 24 | 0.438 |
| CAT | Mean based | 2.164 | 7 | 24 | 0.075 |
|  | Median based | 1.054 | 7 | 24 | 0.422 |
|  | Based on median and with adjusted freedom | 1.054 | 7 | 7.649 | 0.468 |
|  | Based on the clipped average | 1.918 | 7 | 24 | 0.111 |
| GSH | Mean based | 0.218 | 7 | 24 | 0.977 |
|  | Median based | 0.235 | 7 | 24 | 0.972 |
|  | Based on median and with adjusted freedom | 0.235 | 7 | 21.083 | 0.972 |
|  | Based on the clipped average | 0.225 | 7 | 24 | 0.975 |
| Chl a | Mean based | 3.106 | 7 | 24 | 0.018 |
|  | Median based | 1.74 | 7 | 24 | 0.147 |
|  | Based on median and with adjusted freedom | 1.74 | 7 | 7.313 | 0.236 |
|  | Based on the clipped average | 2.819 | 7 | 24 | 0.027 |
| Chl b | Mean based | 4.472 | 7 | 24 | 0.003 |
|  | Median based | 3.923 | 7 | 24 | 0.005 |
|  | Based on median and with adjusted freedom | 3.923 | 7 | 13.251 | 0.016 |
|  | Based on the clipped average | 4.464 | 7 | 24 | 0.003 |
| Chl a + b | Mean based | 8.998 | 7 | 24 | 0 |
|  | Median based | 1 | 7 | 24 | 0.455 |
|  | Based on median and with adjusted freedom | 1 | 7 | 3 | 0.553 |
|  | Based on the clipped average | 6.759 | 7 | 24 | 0 |
| Carotenoids | Mean based | 1.787 | 7 | 24 | 0.137 |
|  | Median based | 1.705 | 7 | 24 | 0.155 |
|  | Based on median and with adjusted freedom | 1.705 | 7 | 16.608 | 0.176 |
|  | Based on the clipped average | 1.786 | 7 | 24 | 0.137 |

Note: Please see Table S2 for traits abbreviations
